# Genome-Wide Identification and Expression Analysis of the *CaNAC* Family Members in Chickpea during Development, Dehydration and ABA Treatments

**DOI:** 10.1371/journal.pone.0114107

**Published:** 2014-12-05

**Authors:** Chien Van Ha, Maryam Nasr Esfahani, Yasuko Watanabe, Uyen Thi Tran, Saad Sulieman, Keiichi Mochida, Dong Van Nguyen, Lam-Son Phan Tran

**Affiliations:** 1 Signaling Pathway Research Unit, RIKEN Center for Sustainable Resource Science, 1-7-22, Suehiro-cho, Tsurumi, Yokohama, 230-0045, Japan; 2 National Key Laboratory for Plant Cell Technology, Agricultural Genetics Institute, Vietnamese Academy of Agricultural Science, Pham-Van-Dong Str., Hanoi, 100000, Vietnam; 3 Department of Biology, Lorestan University, Khorramabad, Iran; 4 Department of Agronomy, Faculty of Agriculture, University of Khartoum, 13314, Shambat, Khartoum North, Sudan; 5 Biomass Research Platform Team, Biomass Engineering Program Cooperation Division, RIKEN Center for Sustainable Resource Science, 1-7-22, Suehiro-cho, Tsurumi, Yokohama, 230-0045, Japan; Key Laboratory of Horticultural Plant Biology (MOE), China

## Abstract

The plant-specific NAC transcription factors (TFs) play important roles in regulation of diverse biological processes, including development, growth, cell division and responses to environmental stimuli. In this study, we identified the members of the NAC TF family of chickpea (*Cicer arietinum*) and assess their expression profiles during plant development and under dehydration and abscisic acid (ABA) treatments in a systematic manner. Seventy-one *CaNAC* genes were detected from the chickpea genome, including 8 membrane-bound members of which many might be involved in dehydration responses as judged from published literature. Phylogenetic analysis of the chickpea and well-known stress-related *Arabidopsis* and rice NACs enabled us to predict several putative stress-related CaNACs. By exploring available transcriptome data, we provided a comprehensive expression atlas of *CaNAC*s in various tissues at different developmental stages. With the highest interest in dehydration responses, we examined the expression of the predicted stress-related and membrane-bound *CaNAC*s in roots and leaves of chickpea seedlings, subjected to well-watered (control), dehydration and ABA treatments, using real-time quantitative PCR (RT-qPCR). Nine-teen of the 23 *CaNAC*s examined were found to be dehydration-responsive in chickpea roots and/or leaves in either ABA-dependent or -independent pathway. Our results have provided a solid foundation for selection of promising tissue-specific and/or dehydration-responsive *CaNAC* candidates for detailed *in planta* functional analyses, leading to development of transgenic chickpea varieties with improved productivity under drought.

## Introduction

Chickpea (*Cicer arietinum* L.) is one of the major legume crops cultivated throughout the world, especially in the Afro-Asian countries, providing great supplies of protein-, carbohydrate-, mineral-, vitamin-, and health-promoting fatty acid-rich food for human consumption [1]. A number of chickpea by-products, such as low-grade and culled chickpeas, chickpea hay and straw and chickpea pod husks are also widely used for animal feeding [2]–[4]. However, chickpea productivity is severely affected by drought which has made development of drought-tolerant chickpea cultivars is the most important goal in many chickpea research programs [5]–[7].

To cope with drought stress, intensive research has been conducted in recent years in both model and crop plants to discover and elucidate genes and molecular mechanisms that regulate drought responses [8]–[11]. Within the regulatory networks that control the signal transduction from stress signal perception to stress-responsive gene expression, various transcription factors (TFs) and their DNA binding sites, the so-called *cis-*acting elements, act as molecular switches for stress-responsive gene expression, enabling plants adapt better to the adverse stressor [12], [13]. Discovery and genetic engineering of genes encoding novel TFs have the potential to develop transgenic crop plants with superior yield under stress conditions.

The plant-specific NAC (NAM - no apical meristem, ATAF - *Arabidopsis* transcription activation factor, and CUC- cup-shaped cotyledon) TF family was discovered in *Petunia* more than 18 years ago [14]. Since then an amazingly large number of studies have provided evidence for the functions of NAC members in almost every biological process in plants, ranging from lateral root formation [15], embryo development [16], flowering [17], regulation of secondary cell wall synthesis, cell division [18], to biotic and abiotic stress responses [19]–[22]. A typical NAC TF contains a highly conserved N-terminal DNA-binding NAC domain and a variable C-terminal transcriptional regulatory region (TRR) that can serve as either a transcriptional activator or a repressor [19], [21]. Several *cis*-motifs have been identified as DNA-binding sites for the NAC TFs, including the drought-responsive NAC recognition sequence (NACRS) [23], the iron deficiency-responsive *IDE2* motif [24], the calmodulin-binding CBNAC [25], the secondary wall NAC binding element (SNBE) [26], and the 21-bp sequence motif (−83 to −63) in the 35S promoter [15]. Being multiple functional proteins, NAC TFs are also able to mediate protein-protein interactions through their DNA-binding NAC domains [15], [19]. A number of the NAC TFs contain transmembrane (TM) helices (TMHs) in their C-terminal region that are responsible for the anchoring to the plasma membrane [27]. These NAC members are classified as membrane-associated, designated as NTL (NTM1-Like or “NAC with Transmembrane Motif 1”-Like) TFs and are grouped in NTL subfamily. Studies of *NTL* members in *Arabidopsis* and rice (*Oryza sativa*) indicated that the majority of the *NTL* genes are stress-responsive [27], [28].

The advance in genomic sequencing has allowed research community to identify the NAC family members in many sequenced species at genome-wide scale, such as 117 genes in *Arabidopsis*, 151 in rice [29], 163 in poplar (*Populus trichocarpa*) [30], 152 in tobacco (*Nicotiana tabacum*) [31], 152 in maize (*Zea mays*) [32], [33], 147 in foxtail millet (*Setaria italica* L.) [34], 110 in potato (*Solanum tuberosum*) [35], 74 in tomato (*Solanum lycopersicum*) [36], 204 in Chinese cabbage *(Brassica rapa*) [37], 88 in pigeonpea (*Cajanus cajan*) [38] and approximately 200 in soybean (*Glycine max*) [39] of which 152 members were identified with full-length open reading frame (ORF) [40]. Taking the advantage of the available genomic sequence of chickpea [41], [42], in this study we have identified *CaNAC* genes in annotated chickpea genome and provided a nomenclature for all the identified *CaNAC* members. We also carried out sequence alignment and phylogenetic analyses to classify the CaNACs according to their phylogeny. Additionally, we studied the expression patterns of the *CaNAC* genes in various organs under different developmental stages using available transcriptome data. Furthermore, to identify *CaNAC* candidate genes responsive to dehydration/drought in either ABA (abscisic acid)-dependent or independent manner for *in planta* functional studies, we characterized the expression profiles of phylogenetically predicted stress-related *CaNAC* and membrane-bound *CaNAC*/*CaNTL* genes in leaves and roots of chickpea plants treated with dehydration or ABA using real-time quantitative PCR (RT-qPCR). Our results have provided an insight into the regulatory functions of the *CaNAC*s in chickpea, and laid a foundation for in-depth *in planta* functional characterization of selected *CaNAC* genes with the final aim to use them for the improvement of drought tolerance in chickpea by genetic engineering.

## Materials and Methods

### Plant growth, treatments and collection of tissues

Seeds of chickpea (*Cicer arietinum* L.) Hashem “kabuli” cultivar [43] were germinated in pots containing vermiculite and were well-watered and grown under greenhouse conditions (continuous 30°C temperature, photoperiod of 12 h/12 h, 150 µmol m^−2^ s^−1^ photon flux density and 60% relative humidity). For expression profiling of *CaNAC* genes under normal and dehydration stress conditions, 9-day-old chickpea plants were subjected to dehydration, ABA and water (control) treatments for 2 and 5 h as previously described [44]. The relative water content of chickpea seedlings was 55% at 2 h and 33% at 5 h after dehydration. Leaf and root tissues of treated plants were separately collected for expression analysis.

### Identification of the *CaNAC* genes in chickpea

All CaNACs annotated in genotype CDC Frontier, a larger-seeded chickpea “kabuli” cultivar (Ca v1.0) [41] were first collected from PlantTFDB (http://planttfdb.cbi.pku.edu.cn/index.php) [45] and from iTAK (http://bioinfo.bti.cornell.edu/cgi-bin/itak/index.cgi) for manual analysis. The sequences of the *CaNAC* genes and encoded proteins were then individually checked at NCBI (Bioproject: PRJNA175619) [41], using both blastN and blastP to identify the chromosomal location of each *CaNAC* gene. Sequences of *CaNAC* genes were also collected from the genome of the small-seeded “desi” chickpea ICC4958 cultivar (Ca v1.0) (Bioproject: PRJNA78951) available at http://nipgr.res.in/CGAP/home.php for comparison [42]. TMHHM server v2.0 (http://www.cbs.dtu.dk/services/TMHMM/) was applied for prediction of the membrane-bound CaNAC/CaNTL members.

### Phylogenetic analysis

Sequence alignments of NAC proteins from chickpea, *Arabidopsis* and rice were performed with a gap open penalty of 10 and a gap extension penalty of 0.2 to construct the unrooted phylogenetic trees by the neighbor-joining method using MEGA (V6.0) software (http://www.megasoftware.net/) [46]. The confidence level of monophyletic groups was estimated using a bootstrap analysis of 10 000 replicates. Bootstrap values are displayed next to the branch nodes. The alignments were subsequently visualized using GeneDoc (http://www.nrbsc.org/gfx/genedoc/) as presented in Figure S1.

### *In silicon* expression analysis of *CaNAC* genes

Expression data available for each putative *CaNAC* gene were retrieved from the Chickpea Transcriptome Database (CTDB) (http://www.nipgr.res.in/ctdb.html) [47], and used for expression analysis of the *CaNAC*s in different tissues and organs of chickpea during development. Detailed information about sample collections for transcriptome analyses was provided in references [47]–[49]. Briefly, shoots and roots were collected from 15-day-old plants [48], and shoot apical meristem (SAM) was dissected from 21-day-old plants [49] grown in pots containing autoclaved mixture (1∶1) of agropeat and vermiculite in culture room (22±1°C, photoperiod of 14 h). Young leaves, mature leaves, flower buds (FB1-FB4, where FB1, FB2, FB3 and FB4 were 4 mm, 6 mm, 8 mm and 8–10 mm in size, respectively), flowers (FL1-FL4, where FL1 was young flower with closed petals, FL2 was mature flower with partially opened petals, FL3 was mature flower with opened and faded petals and FL4 was drooped flower with senescing petals) and young pods were harvested from field-grown plants [47], [49]. Germinating seedlings (GS) were 5-day-old plants grown on wet Whatman papers in Petri dishes [47].

### RNA isolation, DNaseI treatment, cDNA synthesis

Collected chickpea leaf and root samples were ground into a fine powder using Retsch MM300 shaker and mortar and pestle, respectively. Total RNA was isolated using RNeasy Plant Mini Kit and QIAcube system (Qiagen). Measurement of RNA concentration, DNaseI digestion and cDNA synthesis were performed according to methods published earlier [44].

### RT-qPCR and statistical analyses

Gene-specific primers for selected *CaNAC* genes were designed using the Primer 3 [50] (Table S1). RT-qPCR reactions and data analyses were performed as previously described [51]. The *IF4a* gene was used as a reference gene [52], and the ΔCT method was used to calculate initial amount of target genes [53]. Statistical significance of the differential expression patterns between treatments was determined using the Student's *t*-test (one tail, unpaired, equal variance). For considering a gene as dehydration- or ABA-induced or -repressed, the criterion of minimum 2-fold expression change (at least at one time point) with P-value <0.05 was applied.

## Results and Discussion

### Identification and nomenclature of the *CaNAC* genes in chickpea

To identify all the *CaNAC* genes annotated in the chickpea genome, we first collected all the predicted *CaNAC* genes from PlantTFDB and iTAK. These two databases collected the TF sequences from the annotated genomic sequence (Ca v1.0) of the genotype CDC Frontier, a chickpea “kabuli” cultivar [41]. Next, all the *CaNAC* gene sequences were subjected to a sequence comparison to remove all the overlapped genes to build a list of 71 potential *CaNAC* genes in chickpea (Table S2). Subsequently, the identified *CaNAC* genes were blasted against the assembled "kabuli" genome (Ca v1.0) available at NCBI (Bioproject: PRJNA175619) to identify their chromosomal location of each *CaNAC* gene (Table S2). If the gene annotation predicted several splice variants for a given gene, splice variants encoding the longest open reading frames were selected as representative members as provided in Dataset S1 along with their respective protein sequence.

In addition to the genomic sequence of the larger-seeded chickpea “kabuli” CDC Frontier cultivar, the genomic sequence of the small-seeded “desi” ICC4958 chickpea cultivar was also available from another independent genome sequencing project (Bioproject: PRJNA78951) [42]. Thus, we also searched for the *CaNAC* genes annotated in the “desi” ICC4958 chickpea cultivar. Only 62 *CaNAC*s were found in the “desi” ICC4958 annotated genome, with 9 members less as compared with those identified in “kabuli” CDC Frontier genome (Table S2). This may be due to the fact that a lower number of protein encoding genes (27,571 genes) were annotated in the “desi” chickpea genome [42], in comparison with the “kabuli” chickpea genome (28,269 annotated genes) [41]. Additionally, Jain et al. [42] estimated that chickpea genome might have around 32,000 genes, which is approximately 13–15% more than the number of currently annotated gene models. Therefore, we might expect to identify more *CaNAC*(s) in chickpea genome in future by fine-tuning of the annotation. We proposed a nomenclature to name the identified *CaNAC* members from *CaNAC01* to *CaNAC71* following their chromosomal localization in the “kabuli” chickpea genome and the chromosomal order starting with chromosome 1.

### Chromosomal localization, gene duplication and phylogenetic analyses of CaNAC TFs

**Figure 1:**
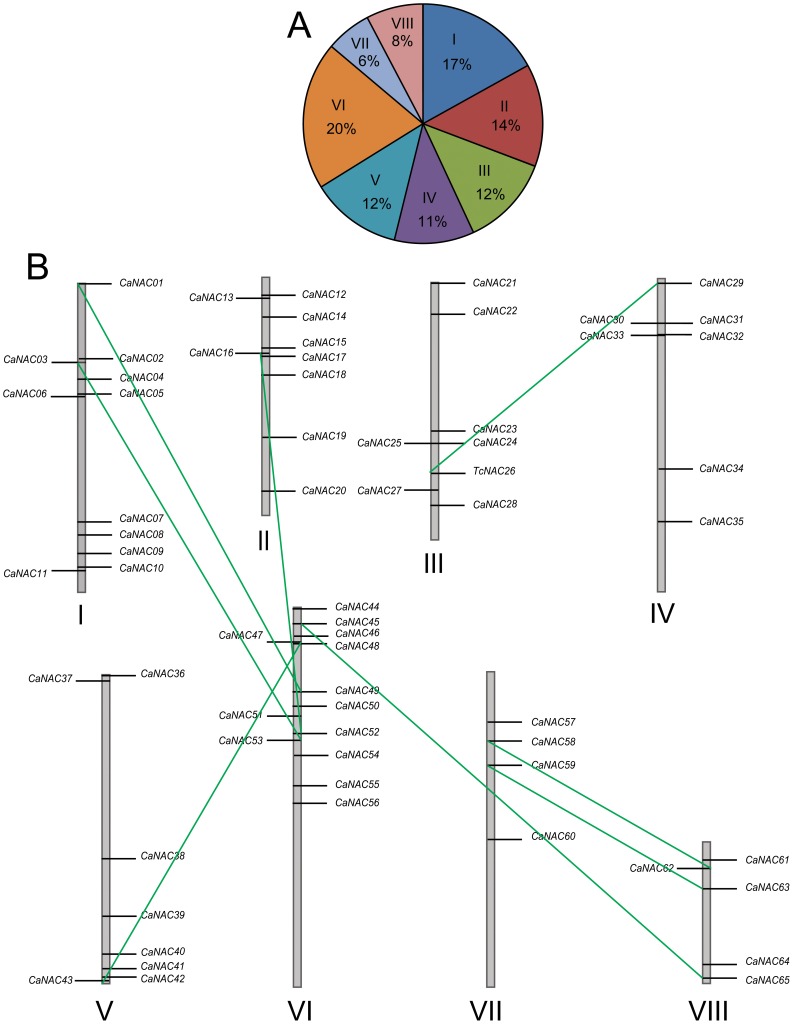
Distribution percentage of 65 chickpea *CaNAC* genes identified in this study in 8 chickpea chromosomes. (A) Chromosomal distribution of *CaNAC* genes with their percentage on each chromosome. (B) Graphical representation for chromosomal localization of *CaNAC* genes. Greek numbers indicate chromosome numbers. Green lines indicate duplicated gene pairs.

Out of 71 identified *CaNAC* genes, 65 members were able to be mapped to the 8 chickpea chromosomes according to the currently available sequence data [41], [42]. These 65 *CaNAC*s are distributed on the 8 chromosomes with an uneven ratio (Figure 1A). The highest number of *CaNAC*s was detected on chromosome VI, with 13 members representing ∼20% of the identified *CaNAC*s, while the lowest number of *CaNAC*s was found on chromosome VII, containing 4 out of 65 mapped *CaNAC* genes (∼6%) (Figure 1A). The exact location site of each *CaNAC* gene is shown in Table S2, and the relative locations of the *CaNAC*s on their respective chromosome are illustrated in Figure 1B. Among the 65 *CaNAC*s, using the criterion >60% homology at nucleotide level we found 8 duplicated pairs, none of which was tandemly duplicated pair (Figure 1B). In comparison with chickpea, in soybean 13 tandemly duplicated clusters of 2 or more *GmNAC* members were identified among 152 examined *GmNAC*s [40]. This observation might suggest the absence of the recent whole genome duplication (WGD) in chickpea, as found in soybean ∼13 million years ago [54]. Indeed, analysis of the rates of synonymous substitution per synonymous site (Ks) within the paralogous gene pairs indicated that the latest WGD event in chickpea was ∼58–60 million years ago [41], [42].

A multiple alignment indicated that all of the CaNACs shared a highly conserved N-terminal DNA binding NAC domain, which consists of five consensus subdomains (A–E), and a variable C-terminal transcriptional regulation domain. Additionally, a conserved bipartite nuclear localization signal was also found in the D subdomain of the majority of CaNACs, suggesting that these CaNACs may be localized to the nucleus (Figure S1) [55]. To examine the structure and phylogenic relationship between the CaNAC TFs and the ANACs of *Arabidopsis*, we constructed a unrooted phylogenic tree based on the alignment of their deduced protein sequences (Figure S2). On the basis of the phylogenetic analysis, we could classify the CaNACs into 12 subgroups together with their ANAC orthologs. This result suggests that the CaNACs are as diverse as the ANACs. On the other hand, increasing evidence has suggested that phylogenetic analysis can be used to predict the function of genes because genes with similar functions are phylogenetically related [32], [40], [56]–[58]. Thus, the phylogenetic analysis also allowed us to predict the function of the CaNACs as many ANACs have known functions [19]–[22], [59].

**Figure 2:**
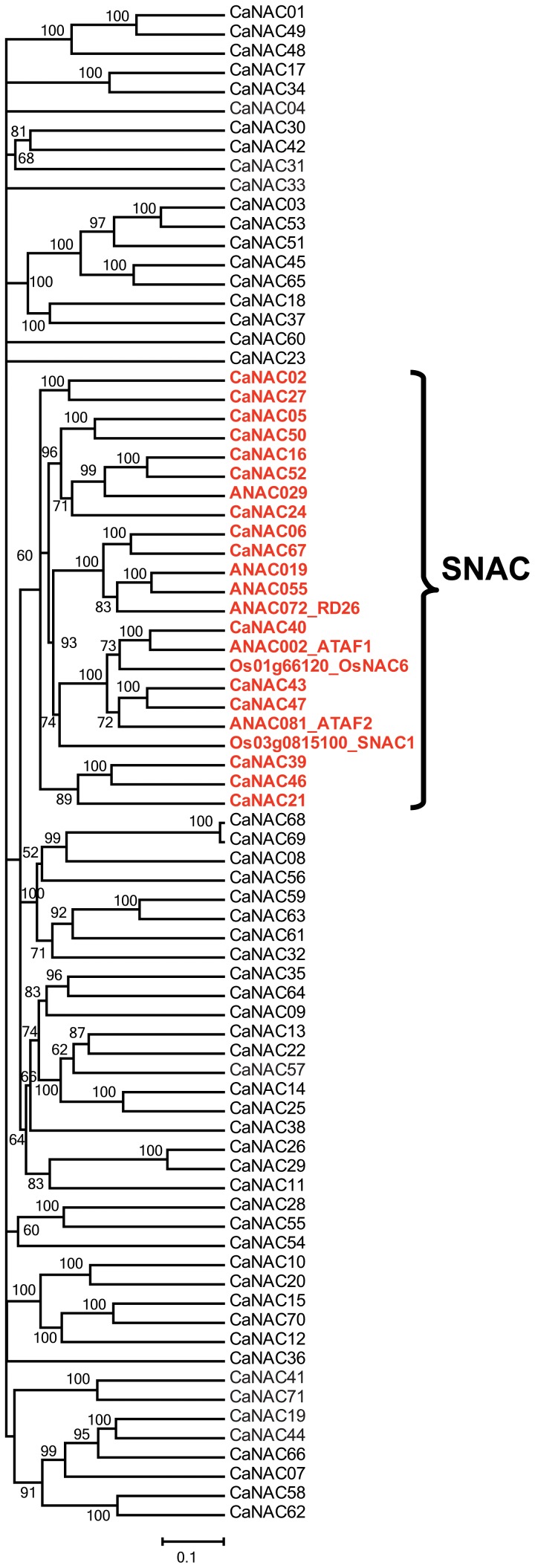
Prediction of stress-responsive *CaNAC* genes based on phylogenetic analysis. The unrooted phylogenetic tree was constructed using the full NAC protein sequences of all 71 *CaNAC* and well-known stress-responsive *NAC* genes from *Arabidopsis* (*ANAC002*, *019*, *029*, *055*, *072* and *081*) and rice (*SNAC1/ONAC002* and *OsNAC6*/*SNAC2*/*ONAC048*). Bootstrap values are displayed next to the branch nodes. SNAC, stress-related *NAC* group.

As we were interested in identifying abiotic stress-, especially drought-related, *CaNAC* genes through phylogenetic analysis for further studies, the most well-known stress-related ANACs and ONACs were selected and included into a phylogenetic analysis of identified CaNACs [23], [60]–[62]. According to the phylogenetic tree shown in Figure 2, by using these 6 stress-related *Arabidopsis* ANACs (ANAC002, 019, 029, 055, 072 and 081) and 2 stress-related rice ONACs (SNAC1/ONAC002 and OsNAC6/SNAC2/ONAC048), 15 *CaNAC*s could be classified as stress-related TFs. Obviously, we cannot rule out that there would be more stress-related *CaNAC* genes, scattered on other branches of the tree, out of 71 identified *CaNAC*s. As evidenced in soybean, when more stress-related ANAC and ONAC proteins were used in phylogenetic analysis-based prediction, more stress-related *GmNAC* genes, clustered into different clades, were predicted [40].

### The membrane-associated CaNAC/CaNTL subfamily

**Figure 3:**
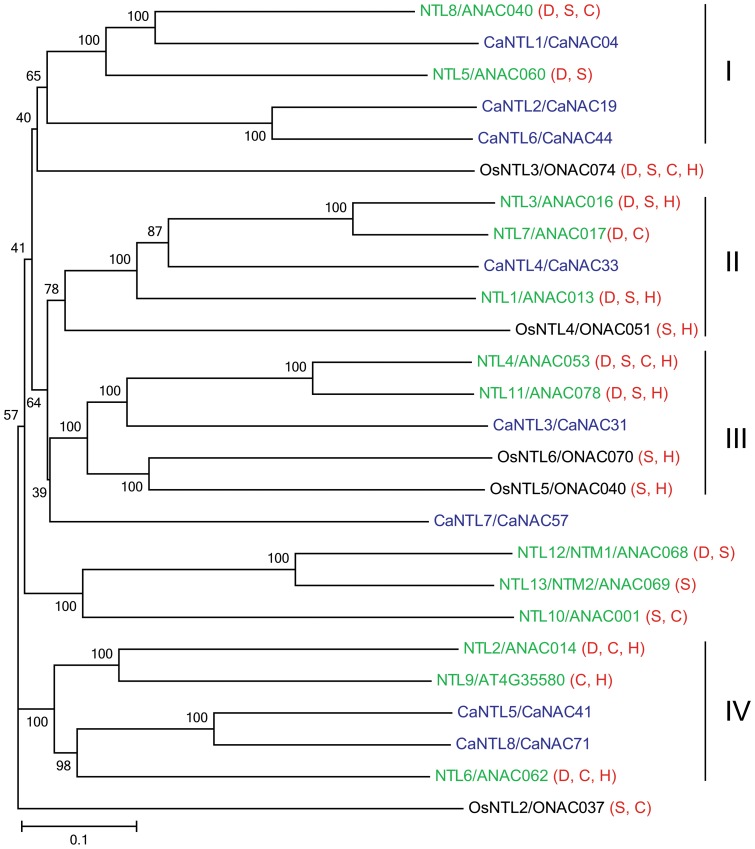
Phylogenetic tree of membrane-bound NACs from chickpea (CaNTLs/CaNACs), *Arabidopsis* (NTLs/ANACs) and rice (OsNTLs/ONACs). The unrooted phylogenetic tree was constructed using the full protein sequences. The bar indicates the relative divergence of the sequences examined and bootstrap values are displayed next to the branch. Stress-responsiveness of each *NTL* gene is shown next to its name in the parentheses. D, dehydration/drought; S, salt stress; C, cold stress; H, heat stress.

Membrane-associated NTL TFs are stored in their dormant form, and when required, their cytoplasmic anchors are degraded, resulting in activated TFs that will then enter the nucleus to regulate expression of target genes [27]. Among 71 CaNACs, 8 members (CaNAC04, 19, 31, 33, 41, 44, 57 and 71) were identified as membrane-associated CaNTLs using the TMHMM v2.0 (Table 1), of which 4 (CaNAC31, 33, 41 and 71) and 4 (CaNAC04, 19, 44 and 57) members contain one and two TMHs, respectively. When compared with NTLs identified in other recently studied plant species, out of 11 putative GmNTLs of soybean 2 members possess two TMHs [40], whereas all the NTLs predicted in *Arabidopsis*, rice, maize, potato, foxtail millet, Chinese cabbage and tomato contain only one TMH [27], [28], [33]–[37], suggesting that the existence of doubled TMHs might be specific to leguminous plants. Interestingly, among 8 CaNTLs, CaNAC57/CaNTL7 contains both of its two TMHs in its N-terminal region. With the exception of SlNAC65 of tomato, which has only a TMH in the N-terminus, none of the other NTLs identified in *Arabidopsis*, rice, maize, potato, foxtail millet, Chinese cabbage and tomato has TMH(s) located in the N-terminus [27], [28], [33]–[37]. As the so-called “Exp number, first 60 AAs” (“Exp”  =  “Expected”) of CaNTL7 provided by the TMHMM server 2.0 was 35.9 (Table 1), much higher than 10, the two predicted TMHs of CaNAC57 might be parts of a signal peptide. In agreement with the unique structure of CaNTL7, a phylogenetic tree constructed from the CaNTLs from chickpea (CaNTLs/CaNACs), *Arabidopsis* (NTLs/ANACs) and rice (OsNTLs/ONACs) indicated that the chickpea CaNTLs were scattered into 4 major groups, whereas the CaNTL7 stayed alone on a distinct branch (Figure 3).

**Table 1:** Putative membrane-bound chickpea CaNTLs.

Gene name	Membrane-bound member	Length (aa)	Transmembrane sequences		Exp number of AAs in TMHs	Exp number, first 60 AAs
*CaNAC04*	CaNTL1	476	331…350	362…384	43.78637	0.00061
*CaNAC19*	CaNTL2	624	535…552	600…622	38.70603	0
*CaNAC31*	CaNTL3	578	553…575		20.90626	0.0002
*CaNAC33*	CaNTL4	558	530…552		21.93664	0.00065
*CaNAC41*	CaNTL5	612	585…607		22.28558	0.00171
*CaNAC44*	CaNTL6	636	539…558	614…633	40.15937	0
*CaNAC57*	CaNTL7	430	15…32	37…59	39.78718	35.9005
*CaNAC71*	CaNTL8	610	582…604		22.60723	0

AA, amino acid; Exp, expected; TMHs, transmembrane helices.

### Expression patterns of *CaNAC* genes in various tissues during development

**Figure 4:**
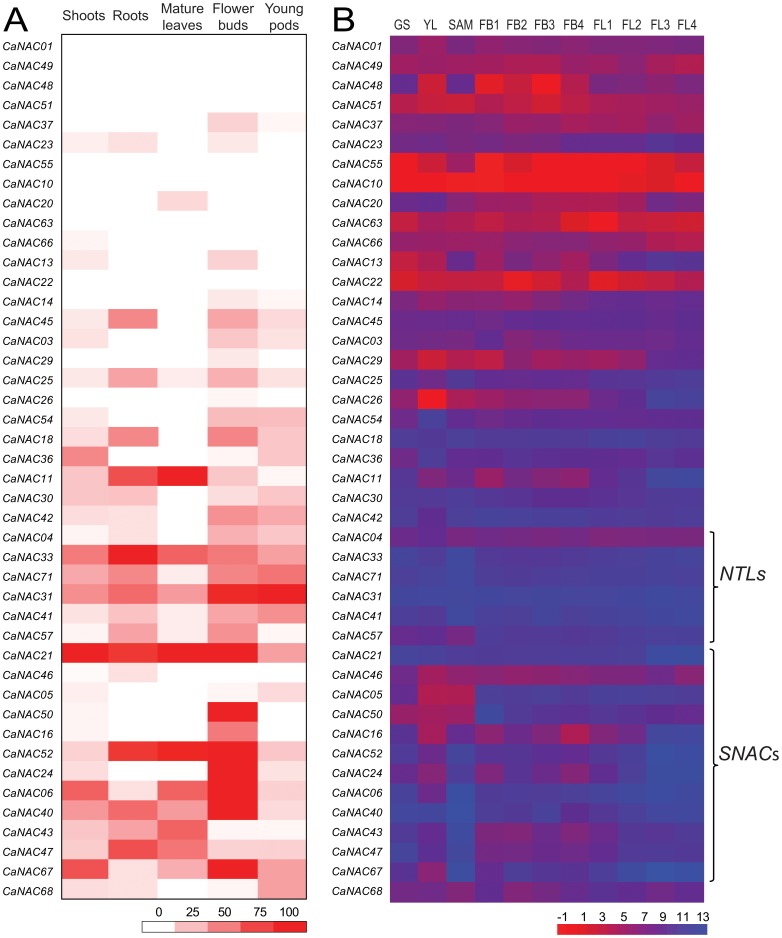
Heatmap representation for expression of *CaNAC* genes in different tissues. (A) The expression data generated by 454 pyrosequencing of cDNA libraries prepared from shoots, roots, mature leaves, flower buds and young pods were obtained from CTDB. Elevated expression levels are indicated by increasing intensities of brown color expressed in RPM (reads per million) values. (B) The expression data generated by Illumina sequencing of RNA-seq libraries prepared from germinating seedling (GS), young leaf (YL), shoot apical meristem (SAM), flower bud stages (FB1-FB4) and flower stages (FL1-FL4) were obtained from CTDB. Blue and red color gradients indicate an increase or decrease, respectively, in transcript abundance represented in log_2_ values. *NTL*s, membrane-bound *CaNAC*s; *SNAC*s, stress-related *CaNAC*s.

Tissue-specific expression profiles are helpful as these data enable us to determine whether a gene of interest plays a role in defining the precise nature and function of given tissue(s). CTDB (www.nipgr.res.in/ctdb.html) provided a comprehensive transcriptome atlas that was generalized for young chickpea seedlings and various types of chickpea tissues collected at various stages of development, including roots, shoots, shoot apical meristem, young leaves, mature leaves, flower buds, flowers and young pods, using either 454 pyrosequencing (Figure 4A) [47] or Illumina sequencing (Figure 4B) [49]. Overall, the expression data for 44 *CaNAC* genes in these tissues could be retrieved from the CTDB, which were presented in a heatmap representation shown in Figure 4. According to the data, the *CaNAC*s possess highly variable transcript abundance. For example, *CaNAC01*, *CaNAC49* and *CaNAC63* exhibited a very weak expression in all the tissues as compared with other *CaNAC*s. The putatively predicted stress-related *CaNAC*s (*SNAC*s) (Figure 2) and the membrane-bound *CaNTL*s (Table 1) are among those with high transcript abundance measured in the tissues. A number of *CaNAC* genes exhibited differential expression patterns being specific in some particular tissues, such as *CaNAC16*, *CaNAC20* and *CaNAC50* (Figure 4A), while many of them appeared to be ubiquitously expressed in the tissues examined across the developmental stages. This phenomenon was also observed for the *NAC* genes in other plants, such as *Arabidopsis*, rice and soybean, suggesting that the functions of the NACs are diversified both in monocotic and dicotic plants [20], [40], [59], [63]. Additionally, increasing evidence has suggested that overexpression of tissue-specifically expressed genes can promote the development of that particular tissue. Transgenic *Arabidopsis* with overexpressed *NAC1* and *AtNAC2* genes, which are preferentially expressed in roots, displayed enhanced lateral root development [15], [64]. Overexpression of the rice *SNAC1* gene, which was induced mainly in guard cells by drought, resulted in an enhanced stomatal function under drought, leading to an increase in drought tolerance [60]. Thus, taken together our results provide a first insight for the readers to link the *CaNAC* genes to their putative *in planta* functions through their temporal and spatial expression patterns.

### Expression patterns of predicted stress-related and membrane-bound *CaNAC* genes in chickpea roots and leaves during dehydration treatment

Previously, through phylogenetic analysis using several well-known stress-related ANAC and ONAC proteins, which are not membrane-bound NTLs, as seed sequences, 15 *CaNAC* genes were predicted to be stress-related (Figure 2). On the other hand, all of the *NAC* genes encoding membrane-bound NTLs in *Arabidopsis* and rice were reported to be induced by at least one type of environmental stresses, namely dehydration/drought (D), salt (S), cold (S) or heat (H) stress as summarized from published literature and visualized in Figure 3 [21], [65]. Thus, in order to identify dehydration-responsive genes for our follow-up *in planta* functional analyses, next we used RT-qPCR to examine the expression of 23 *CaNAC* genes, including all 15 stress-related *CaNAC*s predicted by phylogenetic analysis and all 8 membrane-bound *CaNTL*s, in leaf and root tissues of dehydrated chickpea plants. The expression analyses separately performed with dehydrated chickpea leaves and roots might provide information on the tissue-specific mode of action of the tested *CaNAC*s under dehydration.

**Figure 5:**
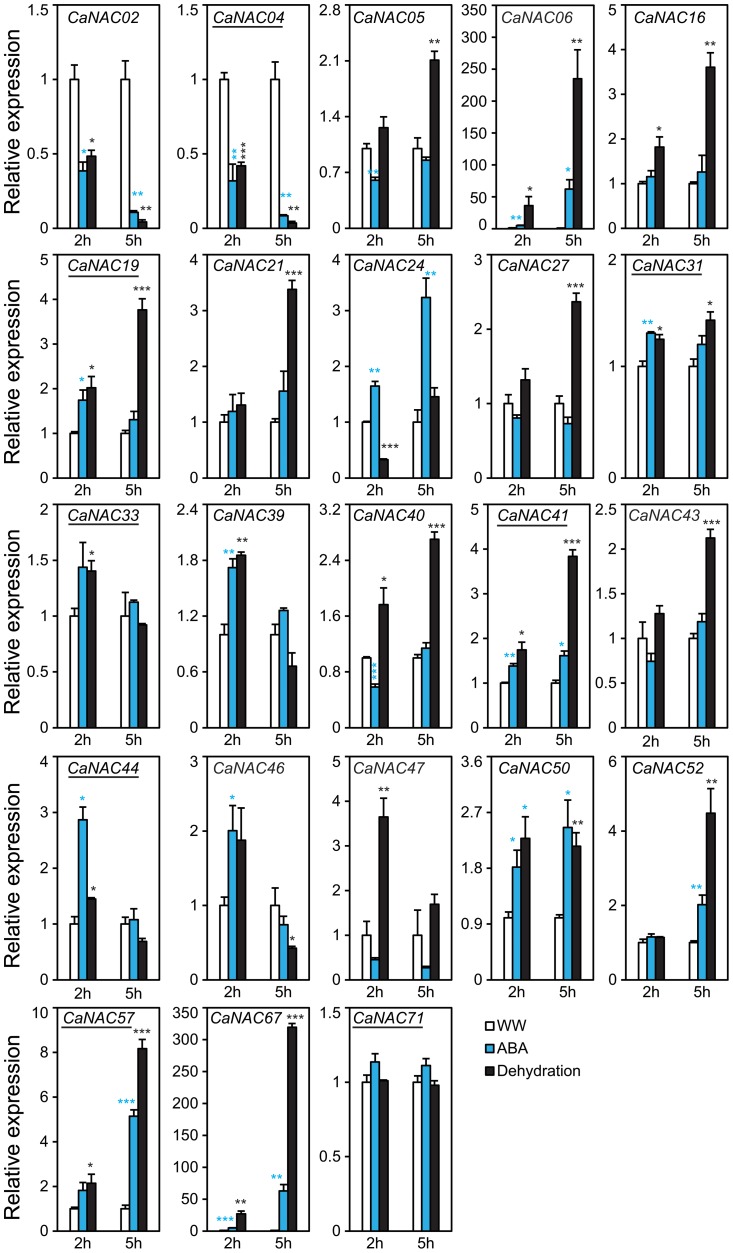
Expression of selected *CaNAC* genes in chickpea leaves under dehydration and ABA treatments. Expression data were obtained by RT-qPCR of treated (ABA or dehydration) and well-watered (WW) control leaf samples collected at indicated time points. Mean relative expression levels were normalized to a value of 1 in water-treated control leaf samples. Error bars  =  SE values of three biological replicates. Asterisks indicate significant differences as determined by a Student's *t*-test (*P<0.05; **P<0.01; ***P<0.001). Membrane-bold *CaNAC*s are underlined.

**Figure 6:**
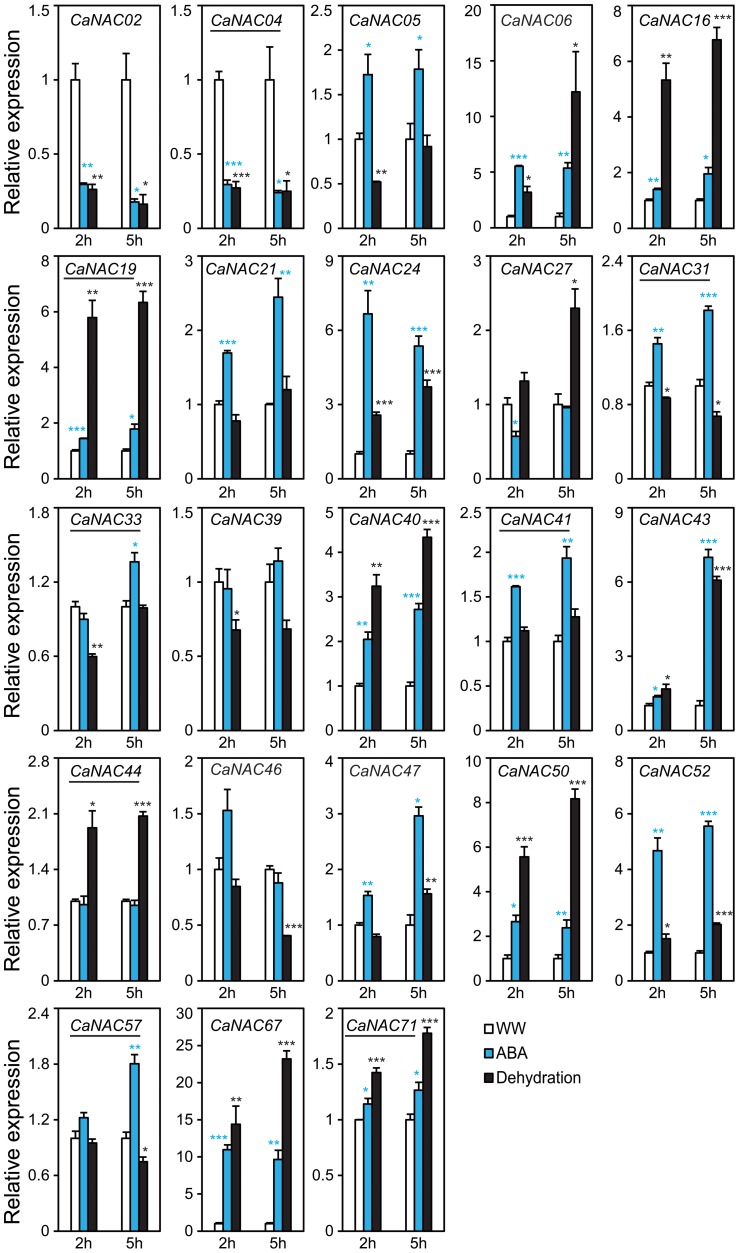
Expression of selected *CaNAC* genes in chickpea roots under dehydration and ABA treatments. Expression data were obtained by RT-qPCR of treated (ABA or dehydration) and well-watered (WW) control root samples collected at indicated time points. Expression data were obtained by RT-qPCR of collected root samples. Mean relative expression levels were normalized to a value of 1 in water-treated control root samples. Error bars  =  SE values of three biological replicates. Asterisks indicate significant differences as determined by a Student's *t*-test (*P<0.05; **P<0.01; ***P<0.001). Membrane-bold *CaNAC*s are underlined.

Using the criterion of fold-change ≥2 and P<0.05, the majority of the tested *CaNAC*s were found to be dehydration-responsive in leaves and/or roots of chickpea plants (Figure S3A). Among the 23 *CaNAC*s, 14 genes, of which 3 *CaNTL*s (*CaNTL2*/*CaNAC19*, *CaNTL5*/*CaNAC41* and *CaNTL7*/*CaNAC57*), were up-regulated, whereas only 4 genes, of which one *CaNTL* (*CaNTL1*/*CaNAC04*), were down-regulated by at least 2-fold in leaves after 2 and/or 5 h of dehydration (Figure 5, Figure S3A). *CaNAC06* and *CaNAC67* were the two most highly induced genes (over 200- and 300-fold, respectively), whereas *CaNAC02* and *CaNAC04* were the two most significantly repressed genes (23.8-fold and 28.6-fold, respectively after 5 h of dehydration) in chickpea leaves by dehydration. As for the roots, 12 genes, of which 2 *CaNTL*s (*CaNTL2*/*CaNAC19* and *CaNTL6*/*CaNAC44*), were induced, whereas 3 genes, of which one *CaNTL* (*CaNTL1*/*CaNAC04*) were repressed by at least 2-fold after dehydration for 2 and/or 5 h (Figure 6, Figure S3A). In comparison with dehydrated leaves, the degree of induction in dehydrated roots was approximately 10-fold lower, with the highest induction of ∼23-fold recorded for *CaNAC67* at 5 h of dehydration. Thus, *CaNAC67* being induced the most highly in both tissues is a promising candidate gene which deserves further and in-depth *in planta* molecular and functional analyses under drought. A number of studies have indicated that TF encoding genes with high inducibility by stress are preferable for selections of further *in planta* functional studies as they might have potential for development of improved stress-tolerant transgenic plants by overexpression approach [58], [66]. Additionally, the repression degree of *CaNAC*s in dehydrated roots *versus* dehydrated leaves was also lower by approximately 4-fold. For instance, *CaNAC02* was the most highly down-regulated by dehydration in roots with a fold-change of only 6.2. In addition, *CaNAC24* was deserved to be mentioned as this gene was induced (2.6-fold and 3.7-fold at 2 and 5 h after dehydration, respectively) in dehydrated roots but repressed (3-fold at 2 h of dehydration) in dehydrated leaves. It would be then interesting to study how *CaNAC24* is involved in regulation of chickpea responses to drought. We hypothesize that under drought stress the up-regulation of *CaNAC24* in roots might contribute to enhancement of root development, whereas its down-regulation in leaves might contribute to repression of leaf and/or shoot growth. These changes would enhance the adaptation of chickpea plants under limited water conditions.

Our data together indicated that out of 23 *CaNAC*s examined, 19 genes were dehydration responsive in roots and/or leaves of young chickpea seedlings (Figure S3A). Of 8 membrane-bound *CaNAC*s, 5 genes were determined as dehydration-responsive, representing 62.5% of the *CaNTL*s and almost reaching to the percentage of *Arabdopsis NTL* genes identified as responsive to dehydration/drought (10/13 genes, i.e. 76.9%) (Figure 3) [21], [65]. A Venn diagram analysis indicated that the majority of dehydration-responsive *CaNAC*s are overlapped in roots and leaves, with 10 and 3 genes up-regulated and down-regulated in both two organs, respectively (Figure S3B, left panel). Three genes, *CaNAC05*, *21* and *57*, were induced only in dehydrated leaves, while 2 gene (*CaNAC24* and *44*) were specifically up-regulated in dehydrated roots only under our experimental conditions. As for down-regulation, only *CaNAC24* was found to be specifically down-regulated in leaf tissues by dehydration (Figures 5–6; Figure S3A). It should also be noticed that out of 15 phylogenetically predicted stress-related *CaNAC*s (Figure 2), 14 genes are dehydration-responsive (Figure S3A), demonstrating that the phylogenetic analysis-based method has an accuracy rate of 93.33%; a quite good rate for a prediction.

### Expression patterns of predicted stress-related and membrane-bound *CaNAC* genes in chickpea roots and leaves under ABA treatment

It is well-established that the NAC TFs can regulate plant responses to water stress through either ABA-dependent or -independent manner [19], [22]. Thus, it was of interest to examine the expression of the selected 23 *CaNAC*s in both roots and leaves treated with ABA. Results indicated that a total of 12 of 23 examined *CaNAC*s were responsive to ABA as their expression levels were altered by at least 2-fold at a P-value <0.05. Out of these, 7 and 8 *CaNAC*s were up-regulated, whereas only 2 and 2 *CaNAC*s were down-regulated in ABA-treated leaves and roots, respectively (Figures 5–6; Figure S3A). Similar to dehydration, ABA treatment also resulted in a significant overlap among the ABA-responsive *CaNAC* genes detected in roots and leaves. Out of 12 *CaNAC* genes responsive to ABA in leaves and/or roots, 5 and 2 were found to be ABA-induced and -repressed, respectively, in both organs (Figure S3B, right panel). Additionally, according to our data, the majority of dehydration-related CaNACs identified in this present study may regulate drought-responsive responses in chickpea in an ABA-dependent manner. Out of the 19 *CaNAC*s responsive to dehydration in leaves and/or roots, 7 genes were recorded as ABA-independent, whereas 12 *CaNAC*s were identified as ABA-dependent. Four *CaNAC* genes (*CaNAC31*, *CaNAC33*, *CaNAC39* and *CaNAC71*) were not responsive to either ABA or dehydration (Figures 5–6, Figure S3A). Our data also suggested that *CaNAC02* and *CaNAC67*, showing the highest down- and up-regulation, respectively, in both roots and leaves by dehydration, act in dehydration/drought responses in an ABA-dependent pathway. In addition to dehydration-inducible promoters [67], ABA-inducible promoters, when coupled with dehydration-inducible genes, are also useful in biotechnological applications for enhancing drought tolerance of transgenic plants [68].

## Conclusions

Research efforts on identification and characterization of the NAC TFs using high-throughput genomic surveys and expression analyses will undoubtedly describe key features of the members of this novel plant-specific TF family. As a result, our current understandings of the regulatory functions of the NAC TFs in various plant species will be definitely accelerated. Our current study, which reported the comprehensive identification and characterization of the *CaNAC* family in chickpea, has provided an insight into the functional diversity of the CaNAC family. Furthermore, our expression analyses of a number of *CaNAC* genes during development, dehydration and ABA treatments have established a solid foundation for chickpea scientists to select candidate genes and their associated tissue-specific and/or dehydration- and/or ABA-responsive promoters for follow-up *in planta* functional analyses, leading to engineered chickpea cultivars with enhanced drought tolerance.

## Supporting Information

Figure S1 **Multiple alignment of 71 CaNACs of chickpea and well-known stress-responsive NACs from** ***Arabidopsis*** **(ATAF1/ANAC002, 019, 029, 055, 072 and ATAF2/081) and rice (SNAC1/ONAC002 and OsNAC6/SNAC2/ONAC048).** Conserved NAC domain and subdomains (A–E) are indicated by thick blue line and black thin black lines, respectively, above the sequences. The putative nuclear localization signal (NLS) is shown by a blue double-headed arrow below the sequence. Putative stress-related NAC subgroup is highlighted in red-colored background, and membrane-bound CaNAC members are highlighted in turquoise-colored background.
(PDF)

Figure S2 **Phylogenetic relationship of NAC proteins from chickpea (CaNACs) and** ***Arabidopsis*** **(ANACs).** The unrooted phylogenetic tree was constructed using the full NAC protein sequences. The membrane-bound CaNAC and ANAC proteins are indicated in blue-colored and green-colored letters, respectively.
(PDF)

Figure S3 **Expression of 23 selected** ***CaNAC*** **genes in chickpea roots and leaves under dehydration and ABA treatments.** (A) Summary of the results of the expression data. (B) Venn diagram analysis of dehydration- and ABA-responsive *CaNAC* genes in roots and leaves of chickpea plants. The ABA- and/or dehydration-responsive genes were defined as those whose expression is altered by at least 2-fold (P<0.05) at 2 and/or 5 h of dehydration and/or ABA treatment.
(PDF)

Table S1 **Primers used in RT-qPCR analysis.**
(XLS)

Table S2 **Putative** ***CaNAC*** **genes identified in this study and their major features.**
(XLS)

Dataset S1 **Nucleotide and amino acid sequences of 71** ***CaNAC*** **genes obtained from (Ca v1.0) (Bioproject: PRJNA175619).**
(TXT)
