# Supplementary material for: Genome-Wide Identification and Expression Analysis of the CaNAC Family Members in Chickpea during Development, Dehydration and ABA Treatments
Source: PLoS One. 2014 Dec 5;9(12):e114107. doi: 10.1371/journal.pone.0114107 (PMC4257607; doi:10.1371/journal.pone.0114107)
Supplement: Figure S1 — Multiple alignment of 71 CaNACs of chickpea and well-known stress-responsive NACs from Arabidopsis (ATAF1/ANAC002, 019, 029, 055, 072 and ATAF2/081) and rice (SNAC1/ONAC002 and OsNAC6/SNAC2/ONAC048). Conserved NAC domain and subdomains (A–E) are indicated by thick blue line and black thin black lines, respectively, above the sequences. The putative nuclear localization signal (NLS) is shown by a blue double-headed arrow below the sequence. Putative stress-related NAC subgroup is highlighted in red-colored background, and membrane-bound CaNAC members are highlighted in turquoise-colored background. (PDF) [file pone.0114107.s001.pdf]

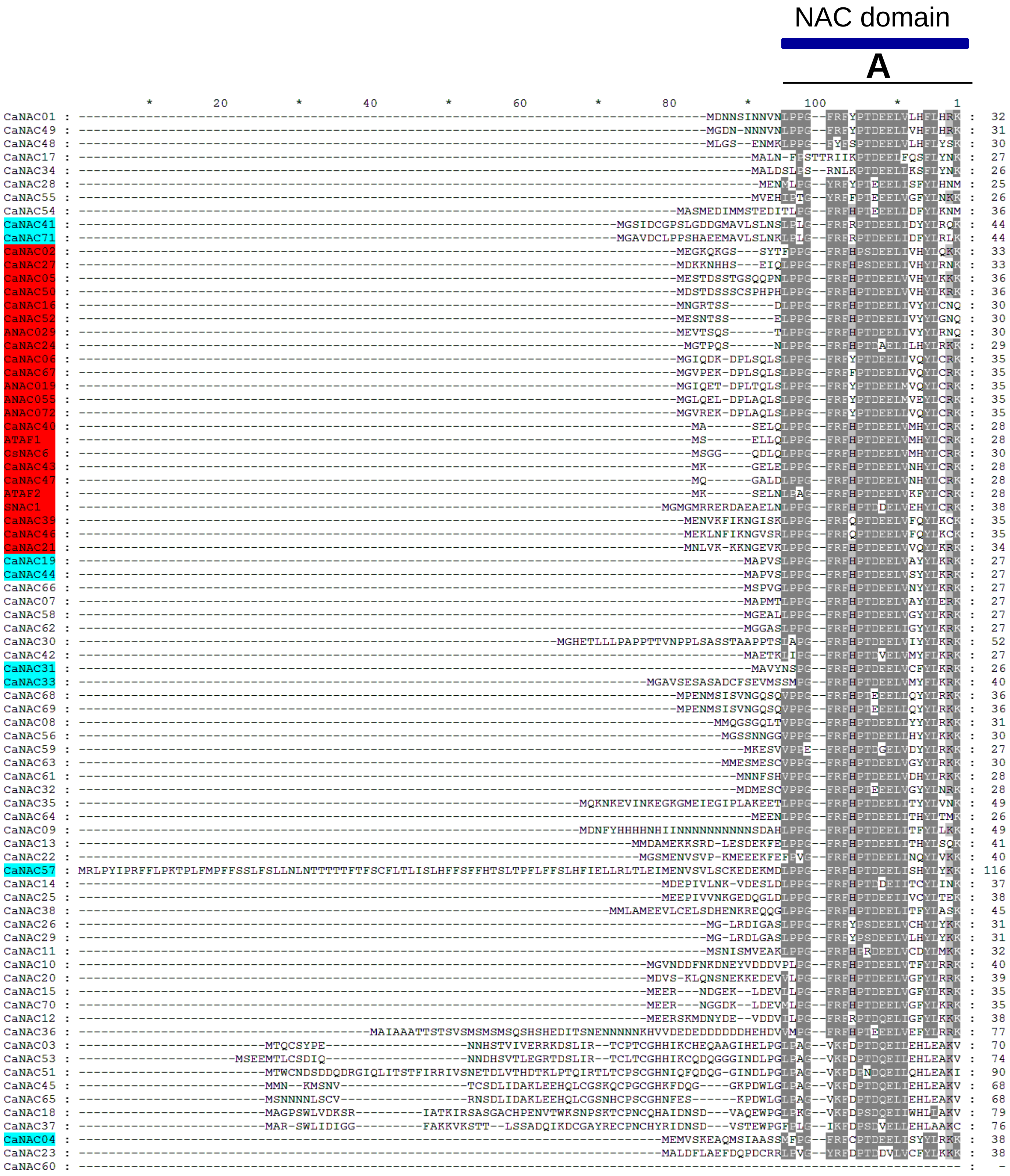

NAC domain
A

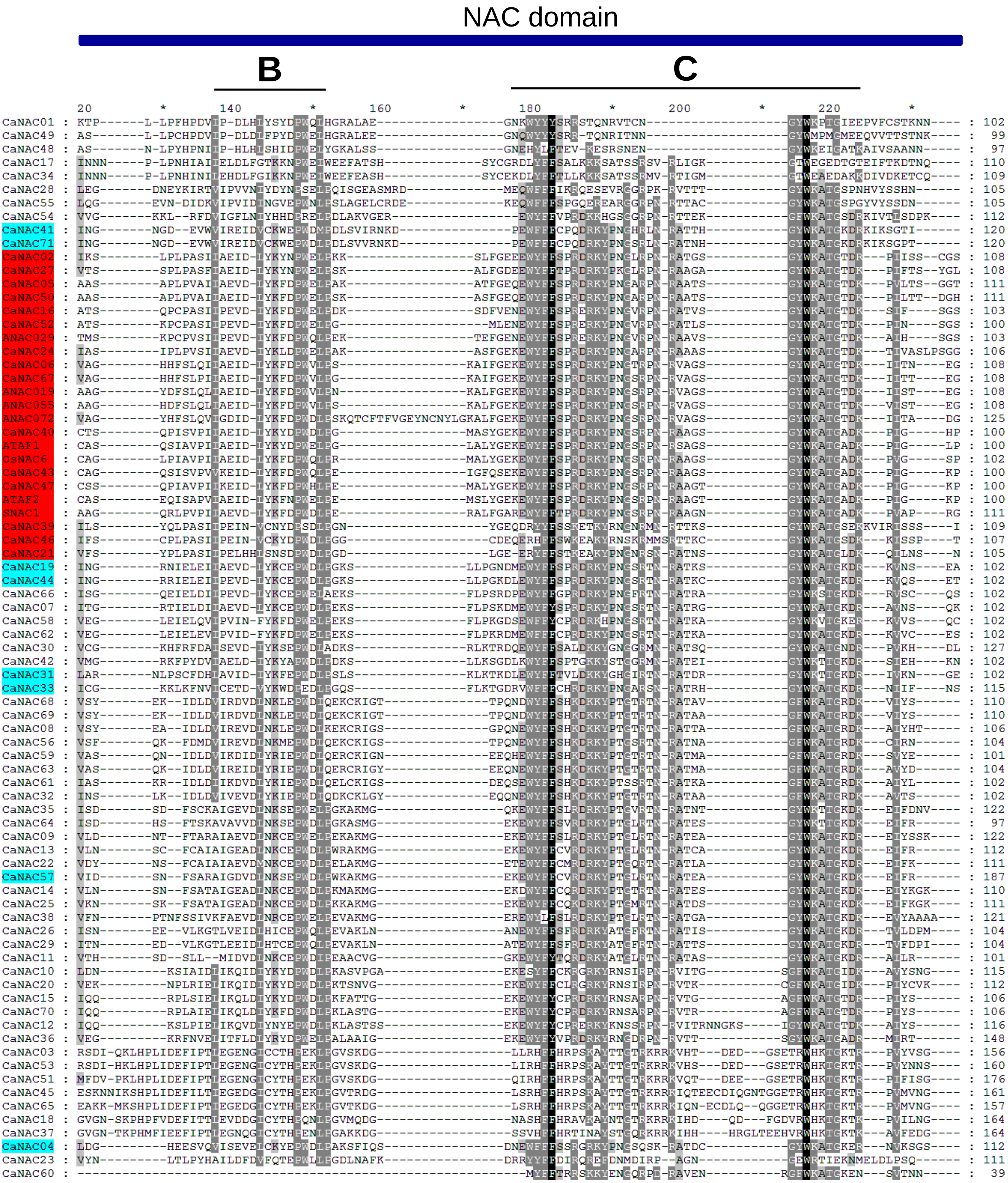
NAC domain
B
C

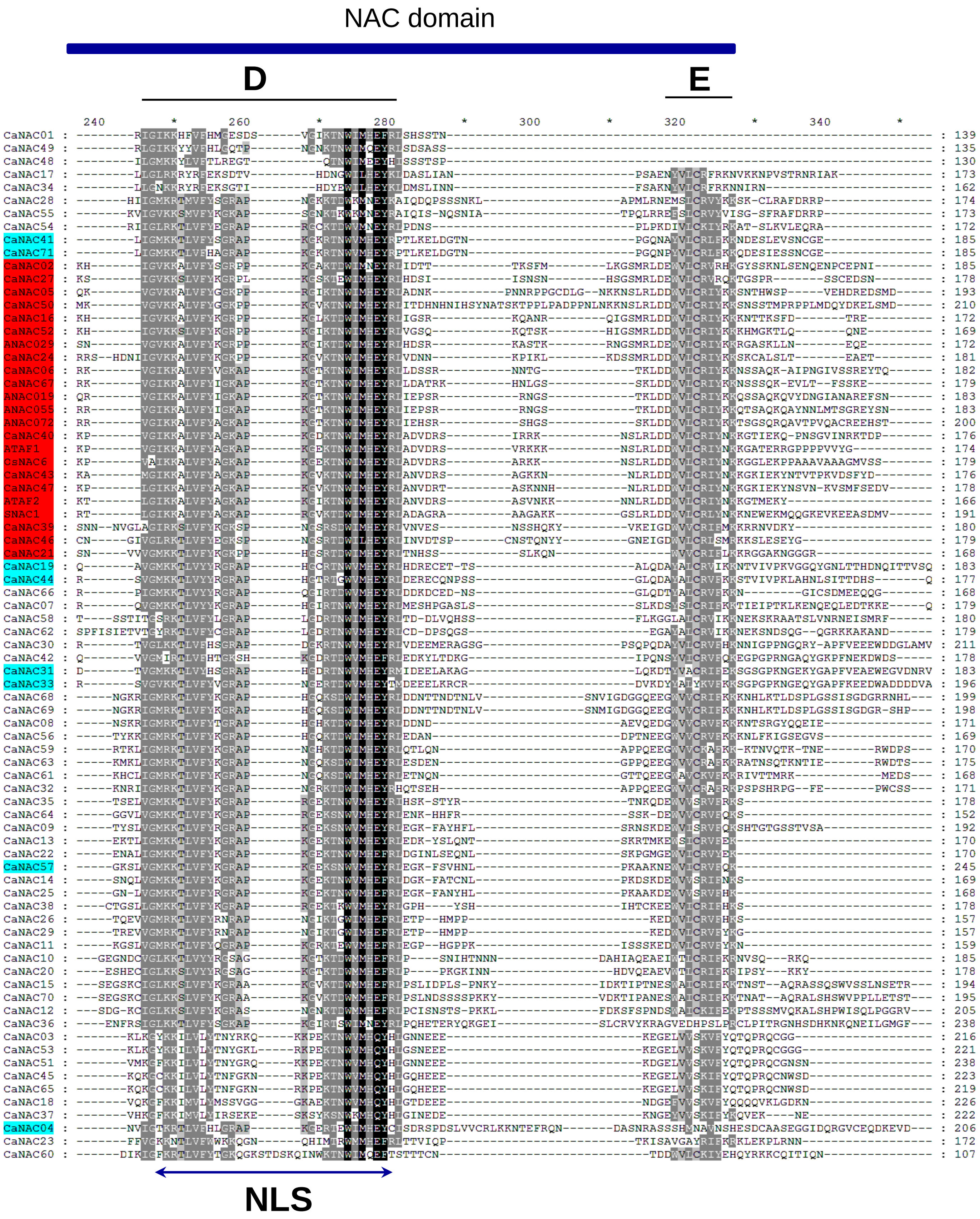
NAC domain
D
E
240
260
280
300
320
340
NLS

```
             360          *       380          *       400          *       420          *       440          *       460          *
CaNAC01 : ---------------------------------------------------------------------------------------------------------------------- :   -
CaNAC49 : ---------------------------------------------------------------------------------------------------------------------- :   -
CaNAC48 : ---------------------------------------------------------------------------------------------------------------------- :   -
CaNAC17 : -----------------------------------------------------------------------------------------------------------------NANRR : 178
CaNAC34 : -------------------------------------------------------------------------------------------------------------------KIK : 165
CaNAC28 : --------------------------------------------------------------------------------------------------------------------AP : 176
CaNAC55 : --------------------------------------------------------------------------------------------------------------------LE : 175
CaNAC54 : --------------------------------------------------------------------------------------------------------------------AE : 174
CaNAC41 : --------------------------------------------------------------------------------------------------------------------VE : 187
CaNAC71 : --------------------------------------------------------------------------------------------------------------------VE : 187
CaNAC02 : ---------------------------------------------------------------------------------------------------------------------P : 186
CaNAC27 : ---------------------------------------------------------------------------------------------------------------------E : 179
CaNAC05 : ----------------------------------------------------------------------------------------------------------------DMIGGV : 199
CaNAC50 : ----------------------------------------------------------------------------------------------------------------HHHT-M : 215
CaNAC16 : ---------------------------------------------------------------------------------------------------------------------E : 173
CaNAC52 : ---------------------------------------------------------------------------------------------------------------------D : 170
ANAC029 : ---------------------------------------------------------------------------------------------------------------------G : 173
CaNAC24 : ----------------------------------------------------------------------------------------------------------------MVGDVD : 187
CaNAC06 : -------------------------------------------------------------------------------------------------------------------YSN : 185
CaNAC67 : -------------------------------------------------------------------------------------------------------------------YSN : 182
ANAC019 : -------------------------------------------------------------------------------------------------------------------NGT : 186
ANAC055 : -------------------------------------------------------------------------------------------------------------------NG- : 185
ANAC072 : -------------------------------------------------------------------------------------------------------------------NG- : 202
CaNAC40 : -------------------------------------------------------------------------------------------------------------------SEI : 179
ATAF1   : -------------------------------------------------------------------------------------------------------------------DEI : 177
CsNAC6  : -------------------------------------------------------------------------------------------------------------------GGG : 182
CaNAC43 : -------------------------------------------------------------------------------------------------------------------HEE : 179
CaNAC47 : -------------------------------------------------------------------------------------------------------------------HFE : 181
ATAF2   : --------------------------------------------------------------------------------------------------------------------FP : 168
SNAC1   : -------------------------------------------------------------------------------------------------------------------TSQ : 194
CaNAC39 : ---------------------------------------------------------------------------------------------------------------------- :   -
CaNAC46 : ---------------------------------------------------------------------------------------------------------------------- :   -
CaNAC21 : ---------------------------------------------------------------------------------------------------------------------- :   -
CaNAC19 : GRGEDLDSSNY-LMQINTCSPHNIGNQISLNRDHGNCSHFSYDDPSFTLPTSSSFTNHGPIAYPPSKVDIALECARMQHRFTMPPLVVEDFPQ-----------GSKTKTDILQEILS : 289
CaNAC44 : GRGDVLDTSNYNFMPCHSTSTQNLHGLLTRD-NNSTWTQFLSDD-LLNLPMSSSFPNYASTPYPPSKVDIALECARMQHRFSMPPLEVEDFPQVSISELNSMCSGNRNETDILQEILS : 293
CaNAC66 : ------------------------------------------------------------------------QCS-----------------------------------------MS : 173
CaNAC07 : GQGEDSSGT---------------------------------------------------------------EISR---------------------------------------EME : 195
CaNAC58 : -SSEPFCKSG---------------------------------------------------------------------------------------------------------DVS : 192
CaNAC62 : -GSNSISTNGN--------------------------------------------------------------------------------------------------------EVS : 192
CaNAC30 : PGAASMNNGSVDRILR----------------------------------------------------------------------------------------------------LE : 229
CaNAC42 : --EEEVDCPQPVPVAA----------------------------------------------------------------------------------------------------VS : 194
CaNAC31 : DPLPAVDNEPLKQIVPSV-------------------------------------------------------------------------------------------PAIDNELLK : 210
CaNAC33 : FKINSPEKEAPNAVIDPL-------------------------------------------------------------------------------------------NGQMQPLFD : 223
CaNAC68 : ---------------------------------------------------------------------------------------------------------------MYDPCDD : 206
CaNAC69 : ---------------------------------------------------------------------------------------------------------------MYDPCDD : 205
CaNAC08 : ---------------------------------------------------------------------------------------------------------------------E : 172
CaNAC56 : ---------------------------------------------------------------------------------------------------------------------- :   -
CaNAC59 : ---------------------------------------------------------------------------------------------------------------NLYDDEE : 177
CaNAC63 : ---------------------------------------------------------------------------------------------------------------YFY--EE : 180
CaNAC61 : ---------------------------------------------------------------------------------------------------------------PCWYDDQ : 175
CaNAC32 : ---------------------------------------------------------------------------------------------------------------SSSQHQH : 178
CaNAC35 : ---------------------------------------------------------------------------------------------------------------------- :   -
CaNAC64 : ---------------------------------------------------------------------------------------------------------------------- :   -
CaNAC09 : --------------------------------------------------------------------------------------------------------------------TT : 194
CaNAC13 : ---------------------------------------------------------------------------------------------------------------------S : 171
CaNAC22 : ---------------------------------------------------------------------------------------------------------------------G : 171
CaNAC57 : ---------------------------------------------------------------------------------------------------------------------S : 246
CaNAC14 : ---------------------------------------------------------------------------------------------------------------------N : 170
CaNAC25 : ---------------------------------------------------------------------------------------------------------------------N : 169
CaNAC38 : ---------------------------------------------------------------------------------------------------------------------- :   -
CaNAC26 : ---------------------------------------------------------------------------------------------------------------------K : 158
CaNAC29 : ---------------------------------------------------------------------------------------------------------------------K : 158
CaNAC11 : ---------------------------------------------------------------------------------------------------------------------R : 160
CaNAC10 : --------------------------------------------------------------------------------------------------------------------QI : 187
CaNAC20 : --------------------------------------------------------------------------------------------------------------------TP : 180
CaNAC15 : ---------------------------------------------------------------------------------------------------------------TSMLTKN : 201
CaNAC70 : ---------------------------------------------------------------------------------------------------------------SNLLTND : 202
CaNAC12 : ---------------------------------------------------------------------------------------------------------------SELLTQT : 212
CaNAC36 : ---------------------------------------------------------------------------------------------------------------VGGQSNK : 245
CaNAC03 : ---------------------------------------------------------------------------------------------------------------------S : 217
CaNAC53 : ---------------------------------------------------------------------------------------------------------------------S : 222
CaNAC51 : ---------------------------------------------------------------------------------------------------------------------S : 239
CaNAC45 : ---------------------------------------------------------------------------------------------------------------------H : 224
CaNAC65 : ---------------------------------------------------------------------------------------------------------------------R : 220
CaNAC18 : ---------------------------------------------------------------------------------------------------------------------E : 227
CaNAC37 : ---------------------------------------------------------------------------------------------------------------------E : 223
CaNAC04 : ---------------------------------------------------------------------------------------------------------------GSSKRGS : 213
CaNAC23 : --------------------------------------------------------------------------------------------------------------------II : 174
CaNAC60 : -------------------------------------------------------------------------------------------------------------------NQN : 110
```

```
               480        *        500        *        520        *        540        *        560        *        580        *
CaNAC01 : SSSSSSKIKSQPIKDYSKWVICRV--------------------------------------------------YESKG--------------------------------------- : 168
CaNAC49 : SRSSSSKRKSQPKSEHSRWVICRV--------------------------------------------------YESDD--------------------------------------- : 164
CaNAC48 : INLEES---------WSKWVLCKV--------------------------------------------------YEKKMSQMSQ---------------------------------- : 155
CaNAC17 : LQRNNTSMGNTGRGRKRKKTVAEC--------------------------------------------------EATTISGNNNNNVVVREYTIYEEPI------------------- : 227
CaNAC34 : MPRKSTRTKNVGQHRRRK-IIIER--------------------------------------------------PETTVSEKNN---------------------------------- : 198
CaNAC28 : RR-----VTNDQEFNKDSTICNHNN-VQIFATSSP------------------------------------------DSS--SSLDHGQTSNEVG----------------------- : 221
CaNAC55 : KERVYELQVRDIDQNQFGATCTHNQAAKMDALSSF------------------------------------------EISQFEGLHSTHMLEDVG----------------------- : 228
CaNAC54 : EEEMKQMVGSPASPPSSTDTISFSCIQQQDQHVSLPMLFLPK------------------------------KESEAETEDMVSLSVSTPSHEKS----------------------- : 239
CaNAC41 : QTTSAPMAANYSPEEIQSDAAPIA-VSTSQVTEDDKHQNVIP------------------------------EISEETISNVIASVDCHSDGYDA----------------------- : 251
CaNAC71 : QTASTPMTANYSPEEIQSDPNVVPGASSSQVTEDYKHLAAIP------------------------------ENSEEAISNFLTPADCYRDACNA----------------------- : 252
CaNAC02 : VNLQTS-EEHPTNTKFRADMITDY------------------------------------------------QYK--DYQIIASILVGGVIPPNE----------------------- : 230
CaNAC27 : LSFETSSHFQQMNDNSSPEPVKNY------------------------------------------------VQKQNEYPMLPYILASKSVLPN------------------------ : 225
CaNAC05 : PPSINVGQMNARFHFSKMSNSYNN------------------------------------------------TLLENDQNLLEGMMLSNNNNNNN----------------------- : 246
CaNAC50 : LPLTNMQQNNTKLPSSRSTSSYG---------------------------------------------------LENDDNFFDGILAADQHQHQH----------------------- : 259
CaNAC16 : YP-TNQINVSPRN-DDDSEQ---------------------------------------------------------ELMKFS------RTCSLT----------------------- : 203
CaNAC52 : YP-TLQFTDSTIVSNNDGEQ---------------------------------------------------------ETMNLP------RTCSLT----------------------- : 201
ANAC029 : FMDEVLMEDETKVVVNEAERRTEE------------------------------------------------EIMMMTSMKLP------RTCSLA----------------------- : 214
CaNAC24 : QAKETQIKDTLFQITKNSTSPNQN------------------------------------------------TLMSQKSMSFSNLLDAMDYSMLS----------------------- : 234
CaNAC06 : DSSAS----SSSHLDDVLQSLP---------------------------------------------------EIDDRCFMLPR--VNSLR-TM------------------------ : 221
CaNAC67 : GSSPS----SSSHVDDMVDSFP---------------------------------------------------EIDDRSFALPR--VTSLR-ML------------------------ : 218
ANAC019 : SSTTS----SSSHFEDVLDSFHQ--------------------------------------------------EIDNRNFQFSN--PNRIS-SL------------------------ : 223
ANAC055 : SSTSS----SSHQYDDVLESLH---------------------------------------------------EIDNRSLGFAAGSSNALPHSH------------------------ : 224
ANAC072 : -SSSS----SSSQLDDVLDSFP---------------------------------------------------EIKDQSFNLPR---------------------------------- : 230
CaNAC40 : EDKKPEILTRGSGLPPHPPPQ-------------------------------------------ATAGMRDYMYFDT-SDSIPKLHT-DSSCS------------------------- : 227
ATAF1   : MEEKP----KVTEMVMPPPPQ-------------------------------------------QTS---EFAYFDT-SDSVPKLHTTDSSCS------------------------- : 219
CsNAC6  : VQRKP--MVGVNAAVSSPPEQKPVVA--------------------------------------GPAFPDLAAYYDRPSDSMPRLHA-DSSCS------------------------- : 234
CaNAC43 : HERKP----EIDKLG--NYQL----------------------------------------------------YMDSSDS-IPRLHT-DSCSS------------------------- : 212
CaNAC47 : NEKKP----QIKMNGQDDFRIN--------------------------------------------------DHTDTSDS-VPRFHT-DSSCS------------------------- : 218
ATAF2   : ADEKP----RTTTMAEQSSSP-----------------------------------------------------FDTSDSTYPTLQE-DDSSSSGG---------------------- : 206
SNAC1   : SHSHTHSWGETRTPESEIVDND------------------------------------------------PFPELDSFPAFQPAPPPATAMMVP------------------------ : 240
CaNAC39 : ----------SVSIMNQPR--------------------------------------------------------------------------------------------------- : 189
CaNAC46 : ----------STSTHHAPSKL------------------------------------------------------------------------------------------------- : 190
CaNAC21 : ------GGENTVTVREKPSKVKNS---------------------------------------------------NSNSNSKV----------------------------------- : 194
CaNAC19 : HTHASQELMNQSNYSQAYDDNENYAPCEDDFTFMVGTNYNHIND-------------------LNSMSYVDKPWEDLSTRSIEIGDQDEEFKEKRMVENLRWIGMPREDFEK---MAE : 385
CaNAC44 : VAQASQDLIHQSNYSHPFGGNQN------DFTFMVGNNYNHVNDH------------------MSSMRYDDKAWEDPNTRSIEIGDLDDEFKAERMVENLRWVGMSSNDLEKSFMEEH : 387
CaNAC66 : LIECSQTIINECETMSPD---------------ILGT----------------------------------------SSSCLEEDDKDDSW--------------------------- : 209
CaNAC07 : NMDHEKVLNNHEHPKFPCDASSS--------DVTQGT----------------------------------------YTPTNTCNNITDDFQP------------------------- : 240
CaNAC58 : SQASHLNN--ESGYSSPVTSPQN------------------------------------------------------VAPIAEFNHVSKETNPS------------------------ : 230
CaNAC62 : SQASQLCSGSESLYSSPIDFSGN------------------------------------------------------VPLMAGFEQASSDTNPS------------------------ : 232
CaNAC30 : GNGGVSCSEGRNNVAQDSQSNNNKAPLDVNKLPIETQNLLAVCKR------------------ESQAEYPSPEKDDSKHMHVDEYPSPQTDNPKPLPQI------------------- : 310
CaNAC42 : APAPILPSSCHISVANDMHPTTS------GCIGLASSSYLSRVMP-------------------SGVTHPSAPSNQVDDDILSMLASFKDDN-------------------------- : 261
CaNAC31 : QIVPVPAASGDDYVEADDLKQELETSVTVGSADPPSNFYYGECSS------------------HPQLSQTFVNDHMQLEASFGMYDPVLDQPDNMAGQYGQDTNLVQDGDYG------ : 304
CaNAC33 : DEIDEIIKEIFDVEPVPDQQYVNGYVDFPQVVGEETQSMILGQFS------------------EAVIFPEASGELQSCSQYYDVQPSFDFNQSIASPLHFSE---------------- : 307
CaNAC68 : EGALEQILRQMGRGCKEENYEANCNNNYERFARPFDHTNLNN-----------------------------MNGGNYNNERFMKLPSLESPKSTSMENNE------------------ : 277
CaNAC69 : EGALEQILRQMGRGCKEENYEANCNNNYGRFARPFDHTNLNN-----------------------------MNGGNYNNERFMKLPSLESPKSTSMENNE------------------ : 276
CaNAC08 : EDHIMNHMRTTNAPCHNNILEPKHHHHKQ-----QQQGLLYD-----------------------------YNNNNNSFDATIHLPQLFSPES------------------------- : 231
CaNAC56 : -THNHQEQQQMNINISSGTNPRSFMHRENQYLLHHQQQQNHS-----------------------------AGFEHEKPELSLHYQHLQQNSQ------------------------- : 232
CaNAC59 : VSGIISRVDPIDLIIRQPQRISAQNF---LYKQEIEPEANN------------------------------LAFMMHSDQLLVPLPQLESPSLQ------------------------ : 238
CaNAC63 : ASGMSSMVDPIDLISRQPQSFLAQSFN--MCKQEIEAENNNNN--------------------------NSLISCMHADQFIQ-LPQLESPSLPL----------------------- : 246
CaNAC61 : VS-LIQELESPNRVSHPYYASYQQNY---PCKQELDQLQYIN--------------------------------ILNDANFLQ-LPQLESPKVPF----------------------- : 233
CaNAC32 : QHQQPHYFRDETYSTTRPLSITDIFHEGTSYSHPFSTEQQQ---------------------------------FLSNQTLIMDKNLIELPQLE------------------------ : 239
CaNAC35 : VGAKKYPSSNPTRATLNPYNINLEVGPSINMQPQPMMHLG----------------------------------DHSTTHFLYGRNYINTPDL------------------------- : 237
CaNAC64 : IASKK-PQQTSSSQPQSPCDTTSIVNEFGDVELHHMDYST----------------------------------NNISPQNFN-TNNVNTNMN------------------------- : 209
CaNAC09 : SGSKKTKITSTTSASNNSMSLCPEPSSPSSVYLPPLLDSSP-----------------------YTAGTAATAFDNRQMCTYNNNNNDNNSSN------------------------- : 264
CaNAC13 : SCGKKMHIQDLVRFNS------IGKELPPLMDSSPNNSELK-----------------------------STIGDSSHVTCFNQNLIEDSQRIQNDN--------------------- : 233
CaNAC22 : DYGKKVHGPKLGRFDSKEGASTTNSLLPPLMDSSLYKSETK-----------------------------FTTGEFSPVNSFSIPNQTQNQKTLDD---------------------- : 238
CaNAC57 : SAGKKTHISGIMRLDTLGN-DLGSSVLPPLTDSSHSIGKIK-----------------------------QLN-DSAYVPCFSN--SIDVQRNQGG---------------------- : 309
CaNAC14 : TEIKKNSFSNLLRINSLSDDLLDFSSLPPLVDPPFS-NTIN--S--------------------PPSSSSKPPPNGNYFPSYLINNNIQNQIP------------------------- : 240
CaNAC25 : TDVKKTQISGLLRMNSINDDLLDYSSLPPLMDPSYTSDELKGIT--------------------NQISSSKSQSDGYYLPSFSINNHNQLVIKPEDHNHRNYDQTQTIMNYTSN---- : 263
CaNAC38 : -VEKRNSIIQVEGGSSNISLKKGSLLPPPPLLPNPTSFSLQ---------------------------------NDQLTHSHFPLHAFQTSFP------------------------- : 237
CaNAC26 : EDNNNNNNSKINTQQLMYETTHPSLTLASSSPTNQTMPSVY-----------------------------------NRIDSFS--SSMTTLHHLI----------------------- : 216
CaNAC29 : TDN----SAKLSPQD-MYETLPPSLTLDSLSPTNQIMPIGY-----------------------------------NQLASFS--SSMTTHHFHN----------------------- : 211
CaNAC11 : EIAAKP-SMGSCYDDTGSSSLPALMDSYISFDQAQFHADEY-----------------------------------EQVPCFSIFSQNQTNPIFNNM--------------------- : 221
CaNAC10 : AEVKQLATKRQMIHDKSSRMSSNVEFNINNQQ---------------------------------------------TFINFGASHENHHNNNI------------------------ : 236
CaNAC20 : NLEKDLATT---VTNSSSSKTCSLESDNSNK----------------------------------------------PYLTFRDSQVVVQQN-------------------------- : 223
CaNAC15 : QETTQFCSNNMS-QTKKTTLPNNFFTNNNNNTQNFDVDVD-VTTP---YKSIINPLLHKAFDHLPISN----NDLNTSLIFSSSPLETSNTNINF----------------------- : 287
CaNAC70 : GNNNQFCSQNMMLLAKKPNFTSQFCATNNHNTTSSNTTLCPLNIPSYNNKPIIDPLIYTPLYKLPISNDQNLNNLSTHGLLFSSPLETSSTNCN------------------------ : 296
CaNAC12 : SNTNQLCSQSNISLSTKTEQESSTIKFCSNNTNNFS-------------PSYFPNYTISKSSQIPNGD----NVVHNNIMLYNTTEATDSTKCP------------------------ : 289
CaNAC36 : AAATPTPTATASINNKMHEGSCSVGNSNSDHVTTVLG---------------LSKYSTDPNYRAAEEE------EEERLMMMQQQQVQYSAGGN------------------------ : 318
CaNAC03 : LMKDSSMIFSDKLNGGQDVHEVVNH----------------------------------------------KNS--GFVEYYNTS-FISFDQGE------------------------ : 262
CaNAC53 : LIKDS--VSEKTIKGQNGNQVVING----------------------------------------------KNSTNGFLDYYRSN-FISFDQG------------------------- : 266
CaNAC51 : IMKDTYENRMSERNVQDDDNIVSKN----------------------------------------------VVP---TMDYYNCDPFMNYEHV------------------------- : 283
CaNAC45 : KSVTNINGEAIEGRRDSGSGSCSNSSKEIVHHINHNHRDEMSAV--------PTPLTNFTSSLD---IQHLKNDHFGFIPFRKSFDEVGIGESCR----------------------- : 308
CaNAC65 : SATTIGEGNGEPNYRDEMSNVNVN--------VNVNG---------------VTPTTSFNHTLDNVQAQQLKSDHFSFIPFRKSFYEVGIGEAST----------------------- : 292
CaNAC18 : LDIPEATEATIIKVDPVTPKSVTPE-------------------------------PPYNERQYSDVDLGQETPAIPQPPQMDCLDEIQADCEEH----------------------- : 291
CaNAC37 : KPLAEEPDNVTSRTSPTTPKPNPPN-------------------------------PPRTGICVENDDNIDETALLLFAEDAKSIPG-ESHAPQS----------------------- : 286
CaNAC04 : SSYGSPSMEQIDSVSESNQRPANDTTLTESSGHLKMDEEDCYAEILKN------------------------DIIKLDESSMSQGAQTRPPDPANSQPCQG----------------- : 290
CaNAC23 : RPVVIDFTMEDATACPPPPPMSP----------------------------------------------------------------------------------------------- : 197
CaNAC60 : VIVSTNIDDTSPNQSNQHFQVQQMQAS--------------------------------------------LSYDMDFTPQYQLSQHFQTQQMQASLLCN------------------ : 166
```

```
                 600         *        620         *        640         *        660         *        680         *        700
CaNAC01 : ---------------------------------------------------------------------------------------------------------------------- :   -
CaNAC49 : ---------------------------------------------------------------------------------------------------------------------- :   -
CaNAC48 : ---------------------------------------------------------------------------------------------------------------------- :   -
CaNAC17 : ---------------------------------------NQSNGGHNSSNDGHNSSMVVKKEYEYKKGVEEEGVKNTCPNDIIVIALESSTDSGNENINQRPRSPIDSGNNKTEVVKT : 306
CaNAC34 : ------------------------------------------------------------------------------------VWLEKNTD-------QRPRNQVDNGNNKIDVVNT : 225
CaNAC28 : ---------------------------------------------EGSQMDINVDHR------------------------EPLLYWEHVDWFLGIEP-------------------- : 250
CaNAC55 : ---------------------------------------------GSSSTNWTLNNNDNDNDN------DNDNDNDELLLQEPLWEWECENFKWP----------------------- : 272
CaNAC54 : ---------------------------------------------TKNQMNGIVKDNNKKGICGTSLQLPFGKDNVPELQLPIATDWTQDTFWAQFNSPWL-----QNWTANSNILNF : 307
CaNAC41 : ---------------------------------------------HDIQNKLAELAAQEDQ-MNFDIYYNPKEGLLDDRLFSPALVHMPQEFHYEANNESDGGCGLQYGTNEINISDF : 323
CaNAC71 : ---------------------------------------------SDAQNQIVTTAAEEDQLFNLDIFDSPQFEQLDDKLFSPVHAHFPQDFFYETNNEVE----FQYGTNETDVSDF : 321
CaNAC02 : --------------------------------------------NMSSLSFKGSKGNNLTSEYEEGSNKVN------SQTAIPSLECYFNP-------------------LKRKSNED : 279
CaNAC27 : -----------------------------------------------SIGMSSNNEKSHASLYENNLNIIG------AQFLSSAAEGLFNP-------------------QKRKAVEE : 271
CaNAC05 : --------------------------------------------TVATLSSQLG----GSASTSNASASKR---TLSSLYWNDHEDVAASN-------------------KRFNLESD : 294
CaNAC50 : --------------------------------------------HHEEMQNGCGSHDINSKNDNNDSFPMKRALTASSQFWNETGSPGSSS-------------------SSKRFHGD : 314
CaNAC16 : --------------------------------------------NLLDMDYLGPISQILSDGSYNSTFEY--------QINTAHGGIIVDP-------------------FVKSQMVE : 250
CaNAC52 : --------------------------------------------YLLDMNYFGP---ILSDG---STFDF--------QINNSN--IGIDP-------------------FVKPQPVE : 240
ANAC029 : --------------------------------------------HLLEMDYMGP----VSHIDNFSQFDH--------LHQPDSESSWFGD-------------------LQFNQ-DE : 256
CaNAC24 : --------------------------------------------SFLSENHSNPSGIGSSTSFNTENFNQP-----SSQINTNNNYMFQKN-------------------TSKQQLSN : 284
CaNAC06 : -------------------------------------------QHRQEEDKLNLLNLNNNNLMDWSNPSS--------ILNT-EFQEGQN-NGMVNYSSCNDLYVPSVSTICHVNTSG : 286
CaNAC67 : -------------------------------------------QQ---EEKLGFHNMDAGIFADWVNPTD--------LESIPEFDSDYNTQGTFNFN-FNDHTVPSVPPFGYMELTT : 281
ANAC019 : -------------------------------------------RPDLTEQKTGFHGLADTSNFDWASFAGNV----EHNNSVPELGMSHV-----------------VPNLEYNCGYL : 277
ANAC055 : -------------------------------------------RPVLTNHKTGFQGLAREPSFDWANLIG--------QNSVPELGLSHN-----------------VPSIRYGDG-- : 272
ANAC072 : ----------------------------------------------MNSLRTILNG-----NFDWASLAG--------LNPIPELAPTNG-----------------LPSYGGYDAFR : 272
CaNAC40 : -------------------------------------------EHVVSPEFAS--EVQSEPK--WNEW-------------EKHLEFPYN----------------------YVDTTL : 263
ATAF1   : -------------------------------------------EQVVSPEFTS--EVQSEPK--WKDWSAV------SNDNNNTLDFGFN----------------------YIDATV : 262
OsNAC6  : -------------------------------------------EQVLSPEFAC--EVQSQPK--ISEWER-------TFATVGPINPAAS----------------------ILDPAG : 276
CaNAC43 : -------------------------------------------GHVVSPDVTCDKEVQSEVK--WNEFGL-------QLD--DAFDFELN----------------------SLLDTS : 254
CaNAC47 : -------------------------------------------EHVVSPDVTCEKEVQSEPK--WNEFVL-------GPDPVSAFDFQLN----------------------FMDDGE : 262
ATAF2   : ------------------------------------------HGHVVSPDV---LEVQSEPK--WGELED-------ALEAFDTSMFGSS----------------------MELLQP : 248
SNAC1   : ------------------------------------------KKESMDDATAAAAAAATIPRNNSSLFVDL------SYDDIQGMYSGLD----------------------MLPPGD : 288
CaNAC39 : -----------------------------------------------FFDFMRVHNAAPGPMITSSSSS-------------TSTEVSS----------------------------- : 218
CaNAC46 : -----------------------------------------------MFDFMMVSNKTCSSTSSSCSSSSI-----------NNIEVSSNA--------------------------- : 223
CaNAC21 : ---------------------------------------------VVFYDFLSSPILSATSGITEEHNENE-----------NENEHEDSS--------------------------- : 229
CaNAC19 : QKVVQIEDISCFQTIREQNGMQ-EYDQHNSNLDVN-----EFSIGFINDGDPNENFIEDGNMDDYSSSPSFYEVIEEIKVSHGLLVSTQQVAMTFFHQVVPSQIVQVQLNPKMIEKNN : 497
CaNAC44 : QKVVPIEDISSFQTNRKENEVQVESEQQNINKELNDNDIENFSMGFINDDDPNENFINEGNIDYSNSTS--YEVVEETKISHGMFVATRQVADTFFHKITPSQTIKVQLNPIME--NK : 501
CaNAC66 : -------------------------------------------MQFITD-------------DAWYS-------------SNAAMVGGEELSHVTFT--------------------- : 237
CaNAC07 : --------------------------------------------QFACDEAN----------SASISYP------MGIGYPSNIFQDIEIPMYGGFHNHIPEAPFMMEDLPQINIMDT : 298
CaNAC58 : --------------------------------------------------------------NFWIS--------------SDMILDSSKE---FTQVKDVVSQYFPRCD-----SPR : 264
CaNAC62 : --------------------------------------------------------------SFWLS--------------PDMILDSSKQDYSQVQVQDVVGSFPQQHDLLSAMTPW : 274
CaNAC30 : -------------------------------YKRRRHNMNSNHSNVSGDSFPNSQDPCSSTITTAATTLPTTAVTTTTTNTAPKKHFLSAL------------VEFSLLESLESKENH : 385
CaNAC42 : -------------------------------------DTSAKNENNGSEKVDNSGQ----------------------ANNAEGAPYLDPN------------EIFESLGDLDS---- : 304
CaNAC31 : ----------ELNLGENPLNFNFASDDPDLYFDATGYLTSLGEEYMETNDVENLDEISPPEIDPSVAAMLDEYLNYPAGDISKHISFDSPLSIGCESPIANPVQPFIEQNVEDEANGS : 412
CaNAC33 : --------------------------------SPELASAPDIESDFQEDGFLEINDLIDTEPTFSNTEKPVENLQFEDGLSEFDLHQDGEMLLRELGPVTGEPVSRAYMNNIGNNIEN : 393
CaNAC68 : -----------------------------NNNNNGYHAIIQENEGSFTNNHNNMVNPLETSSSSTQMVS-------GLTNWVALDRLVASQLNGQTEATRQLACFNDPTIGYCTNDHH : 359
CaNAC69 : -----------------------------NNNNNGYHAIIQENEGSFTNNHNNMVNPLETSSSSTQMVS-------GLTNWVALDRLVASQLNGQTEATRQLACFNDPTIGYCTNDHH : 358
CaNAC08 : -------------------------------------AVPPPPPTSFPSSMDILECSQNLLRLTTPAFS-------GLN----LMQQQGERFHGDWSFLDKLLASHN------GIDHH : 295
CaNAC56 : -----------------------------------YSSLFQSQ--SLLHAQKPLEVNFDYSYASSSLHS-------EQPITVKQLMTNPRDCESGSDGLRYQVSESGIEVGSCEPPRS : 306
CaNAC59 : --------------------------------------SKRQNSMSIISENN--NNDDS-------LYS----------------KIKSKEKVTDWRDLDKFVASQLS-----QEDR- : 287
CaNAC63 : --------------------------------------VKRPSIVSLVSENNDDNNDDDQKR--LLLLSNNNVTTITTTNNNNNNNNNNNQVTTDWRALDKFVASQLS-----HEDEG : 319
CaNAC61 : --------------------------------------QCSSSHNSLIQEQHIQAQQQHCQQQNMQLFS-------------------GRNDVQDWRLLDKFVASQLS-----RDIQD : 289
CaNAC32 : ------------------------------------------ECNQLQHHNNEDCNEER-----------------------------SNNNIVDWKSLDNLFTSQFT-----DTTT- : 280
CaNAC35 : ---------------------------------VEVNRVLRSSGV---VPLPMQYSNL-----SSSPLG--------FTISGLNLN-LGGAS-TQP------STQVHDFSSNMMTT-- : 296
CaNAC64 : ---------------------------------LTMNSWPSSSDVPHSLPISLPWPSS-----LLNPNN--------INVNSLLLKALQLRS-YQQQREVVAPTNIHHFAPYMSQVGV : 280
CaNAC09 : ---------------------------------NTTKEHVSCFSTTSTTSNVVSCHNN-----FNNNNGS-------FDLVSPSMNAMDPFARFQRNVGVSAFPSLRSLQDNLQLP-- : 335
CaNAC13 : -----------------------------IIDSFESPILTSSYS-YSSNPYDNNISSA----SWTQNLSH-------QSTQVVNSKSSDCFMVQEQSMLRMLIEN--HGTSTKTKFEK : 308
CaNAC22 : -----------------------------IVDNNGTSILNISCSSKAIDDYPLAEAAF----AVSNQSSQ-------VAPQIGNPQYSNCFLSQEQSMLRMQLENENLESSAKQCVKA : 316
CaNAC57 : -----------------------------IFDSFTN---NNSIYGVSSNPLISTSSNP----LYSNN-NQ-------VGCNLQFP--SSVCGMQDQAILRALYENNGFKSERQQIVSV : 381
CaNAC14 : ------------------------------SYNIATNQENTFSHQP-QNQIQIQNSSS---QLQQNMLSN-------YSYNMQQGN-HNNTYIYGL---DNKECKMEQFSTNNSMVSV : 313
CaNAC25 : -----------------------------ISNSIQSNLNNPIGNTLSQPQIRIQNQNLNYFVYQQNRMQN-------VNSISSFGNNHDQAFVRPFGAKNNSECKMEQFSSNQ----- : 340
CaNAC38 : ---------------------------------ITHDMSQQNFTLQKEPNIPKTEATF-----YELYQSQ-------SIDDVVNLRWIDHINNFQSSFPLEMNDDDELIAFSS----- : 305
CaNAC26 : -----------------------------------------------------PNQN------NSSMMN--------------LLHYSRDTNPNNNNSTITQISSKGDHDGYGFLW-- : 259
CaNAC29 : -----------------------------------------------------PNVNQ-----NNSLMN--------------LLQFSKETN--TNCSSVTQISPKCD-DGYGFLW-- : 252
CaNAC11 : ------------------------------------------------EPKLPPYNNN-----NNNNANT-------FGGAPNLGNYSLDPLSCDRKVLKAVLTQLSKMERNNPLN-- : 277
CaNAC10 : ----------------------------------------------------EDKLMINTN--------------------YTNIDQ-RNHQLMSCQVSQQPQQNVTLSNSNLWIKQQ : 281
CaNAC20 : ----------------------------------------------------ESKPFING-----------------------QLDQSINYSFLSHVAHEAP---TTLSHSCFWNDHS : 263
CaNAC15 : ----------------------------------------------SKSTMDVSSMLLNMSSSMFVGDFSKTCDEDITTNFNDGLQEHFSDYSTSFLQTTFGNNQYDNNALVKINPYY : 359
CaNAC70 : ----------------------------------------------KTSSMDVSSLLLGMSSSSFL-------EGATTSNYIGSLQDHYNNNNNNGYPIMANNINPNVNVAGVNDEEL : 361
CaNAC12 : --------------------------------------------------IDTSSIISNTN---YIG--------------LEDANNQYSNFSINLPKDIQPNIGTELEEQDWKKNPY : 340
CaNAC36 : -----------------------------------------------------STLLLPLPAACFP---------------LQNSAVSSNSSMMDDLNRLVSFNNNNQHHYCYNPNPN : 368
CaNAC03 : ------------------------------------------------------------QQHRSSN-----------AQVISHFPVHD-----GTP--FIP---------------- : 286
CaNAC53 : ------------------------------------------------------------NQHRDTN-----------AQVINHFPVLE-----GAPNFNIP---------------- : 292
CaNAC51 : ------------------------------------------------------------GHNLESS-----------PQLISNLVMQG-----DDGSSFIRLAMDANKARLDRN--- : 322
CaNAC45 : --------------------------------------------GREIQQASGRCDEHEHEQINATTSFHMSRPSNPISTIISPPPLHHTSIILDDNSYHVSTIMLQNDHFQQQQQQH : 382
CaNAC65 : --------------------------------------------ASEVQAASRSYEEEVHERHKQQN-HHIATS----AFHISPPPLHQTSIILDENSYQVSRIMLQNEHFQQQ--QH : 359
CaNAC18 : --------------------------------------------AIDDLSMIETENNEETYDKEINADEGQKWWDSESQNLLDSQQLVEALSLCDDLLHSQSPERDDEHGEHKNQPSL : 365
CaNAC37 : --------------------------------------------DIQDQ---ENTPNPAWFAGESQAIENSEYDG--LDDILLCKEILDSFALFDDSGLDSITTNDFDYYENKMTGNG : 355
CaNAC04 : ----------------------------------TAQRRIRLRVEKSTHSQPGVRFSRTMHQSKSSSFARMTNHLMIFAFFVIALMVLFFWSLLGGHKQMKYCVKILGVNLVMSRPML : 374
CaNAC23 : ---------------------------------------------------------------------------------------------------------------------- :   -
CaNAC60 : --------------------------------------------MDFTPQCQLNQHFQAQEMQISLSSNIDFMPQYQHNQHFQTQQIQASLPTEIDFTPKYQVNQHFQVQQMQTPLSS : 240
```

```
                *        720         *        740         *        760         *        780         *        800         *        820
CaNAC01 : ----------DDDDDGTDQLSCLDEVFLSSLDDFDEISLPNYN--------------------------------------------------------------------------- : 201
CaNAC49 : ----------DEDGDGT-ELSCLDEVFLS-LDDLDEVSLPN----------------------------------------------------------------------------- : 193
CaNAC48 : -----QVCYSDEDVDSGTEVSWLDEVFMSLDDDLDETSQPN----------------------------------------------------------------------------- : 191
CaNAC17 : TKE-KEVDSIMNKEEDDNLNMSWPEYFAMELKMINEGCLIQEEEYVPMLSNSFHKDLILECFNMY----------------------------------------------------- : 370
CaNAC34 : INTSKSKYNKVEDEEDGDQNIIEPELFAMQLKSVNEGCLIEEED-VPMISNDFYKYLLLENFYIK----------------------------------------------------- : 289
CaNAC28 : ---------------------------------------------------------------------------------------------------------------------- :   -
CaNAC55 : ---------------------------------------------------------------------------------------------------------------------- :   -
CaNAC54 : ---------------------------------------------------------------------------------------------------------------------- :   -
CaNAC41 : LNSDLNWDQLPYVESGCHQQIFSLFNVKENGSGSDSDVEVANMTCMPTTYP-EEAIDRRIPCVTAPESRSTFDCSGDDDRKSKVVLLQNNFQMAYPSDGNIGEVYNVVNGNIGEVYNV : 440
CaNAC71 : FNSAVNWDEVSREASSSLELKSDLLHVKDNGSCSDSEVEMANMMHLQPLHAYPEGVTPTPQNSDAGLFYSSFFSTG---------------PTPTVVDECMGQTPAVVDESMGQTPAV : 424
CaNAC02 : DDQFENLISFTSKFNTENKIDKSFNKVLTNRELNCYEIFNGSTPNPSIDFQELNELNFAE---------------------------------------------------------- : 339
CaNAC27 : NIEMDFYAVLNKNLSRE--VDD-VNQRLEIDLSKGYNSYNFDQWTSIIQPQELNSLAFTGYT-------------------------------------------------------- : 330
CaNAC05 : HHHGSVVRNDQESGTVATSFASLLNNLPQTPNSLHQQTMLGSIGDGLLRTTPYQIQGMNWYG-------------------------------------------------------- : 356
CaNAC50 : LNSG--NSNAEEN----NSFVSLLSQLPTTT-TFHPNAILG---DAVIR-QQFQLPNINWN--------------------------------------------------------- : 364
CaNAC16 : MATNSYAADSEKNN------VKQNSSF---NSTLFGNQVYD---HRE----------------------------------------------------------------------- : 285
CaNAC52 : MTNHFYEADSRKY--------------------------------------------------------------------------------------------------------- : 253
ANAC029 : ILNHHRQAMFKF---------------------------------------------------------------------------------------------------------- : 268
CaNAC24 : IDEDTLLYQSKKYLNSSCNFPNINSQY---ENYLMKQSLLN---QQLILGPHLQFQG------------------------------------------------------------- : 335
CaNAC06 : TEKKPMEEEVQSGGARTN-----PGLFERGSNNFTYSNSVDSFGFRYPVQPVGFGFGQ------------------------------------------------------------ : 339
CaNAC67 : ACRKAAEEEVQSG-MRTQRVTETSGLYQHNSN-IFLSGLGDPFGFNYSGH-------------------------------------------------------------------- : 329
ANAC019 : KTEEEVES--SHGFNN-SGELAQKGYGVD---SFGYSG--QVGGFGFM---------------------------------------------------------------------- : 317
ANAC055 : GTQQQTEG--IPRFNNNSDVSANQGFSVDPVNGFGYSGQ-QSSGFGFI---------------------------------------------------------------------- : 317
ANAC072 : AAEGEAESGHVNRQQNSSGLTQSFGYSSS---GFGVSG--QTFEFRQ----------------------------------------------------------------------- : 314
CaNAC40 : ------NSGFGSQFQ-SNNQMSPLQD------MFMYLP--KTF--------------------------------------------------------------------------- : 291
ATAF1   : ------DNAFGGGG--SSNQMFPLQD------MFMYMQ--KPY--------------------------------------------------------------------------- : 289
CsNAC6  : ------SGGLGGLG--GGGSDPLLQD------ILMYWG--KPF--------------------------------------------------------------------------- : 303
CaNAC43 : NLSLDLDGPFGTNFEYQMNHLSPLQD------MFMYPN--KPF--------------------------------------------------------------------------- : 289
CaNAC47 : ------DDPFAPNAQYQMNQLSGWQD------LLMYLP--K----------------------------------------------------------------------------- : 289
ATAF2   : -------DAFVPQFLYQSDYFTSFQDPPEQKPFLNWSF--APQG-------------------------------------------------------------------------- : 283
SNAC1   : -------DFYSSLFASPRVKGTTPRAG------AGMGM--VPF--------------------------------------------------------------------------- : 316
CaNAC39 : -------SDQTSGYIC------------------------------------------------------------------------------------------------------ : 227
CaNAC46 : -------PDHQQDYTHF----------------------------------------------------------------------------------------------------- : 233
CaNAC21 : -------SSFPSLTNNN----------------------------------------------------------------------------------------------------- : 239
CaNAC19 : HSIKNVEAMMMIMENQG-SLFRKFKAYVMGNLINPSKKIASLVVFLFSLLLMHCVYMEKEVEIWKQNLENVKEKRMRES----YEIIKWNESKEVWFVGVKSEKGLSVVLKKIGIFLT : 610
CaNAC44 : QSIENKEITLIIPKNQGNSLLRKFKANFIGYMKKPTKTMVNAIVFIFALFLVLCVYLKEQVEDWKPKPEYVKRKNCYEASNSMKRVINWNEQEKIWFIGIKSGKGFDVVLKKIGIFLT : 619
CaNAC66 : ---------------------------------------------------------------------------------------------------------------------- :   -
CaNAC07 : KSTLMKPEMIITDDCILYDRYRDCMNGTLEEIISLCSSQDHSMAFPML---------------------------------------------------------------------- : 346
CaNAC58 : QS-EHTTISPSLSYSNFNSEIEFVYNPSQIGYMSPYSNQVNLMDNYGNWNVPYEGYDKLNLVSYQEPF-------------------------------------------------- : 331
CaNAC62 : QSFENTEISPSSSYSNFNAEIEFSDALSRIGCISPYSGQGSFMD------LPYECYDQIHSFGDPKKF-------------------------------------------------- : 336
CaNAC30 : TSVPPVDFDMDSLETSVPPSCAKYIKHLQSEKQKLGVEKETMRFELMSAQAMINILQSRVEIISRENEEMKRMMRNP----------------------------------------- : 462
CaNAC42 : -----------------------LIGLDEGDGFSYGQKDEYTKNEMPSTSNVSLFCDPQHDFLELTDLEAPLF--------------------------------------------- : 354
CaNAC31 : SLANKQVSEAQSSNEAIPTEDPEVSSWVSGDTNPFVKQANKLLASLPAAPAFASEFPTKEFALRIHPAAQSSSSAHITAGMISITDITFRGNAMDWMVGKNGGFNTIMSTGFSQTDGN : 530
CaNAC33 : QSYQLTPYPEDTN----QMVDEFWMHFERNTLSPAEGYADSFSLTNPGVVCDSVSFPSESTENRRSTVEDVSTTSRFSSALWAFVESIPTTPASAAENPLVGRALNRMSS-FSRVKIK : 506
CaNAC68 : DLQFPTLRSSSSTSSISIHAKATTS--TTTSTYITPSQDYTSEIDLWNFARSTSSLLSSSEPICHVSNTSV----------------------------------------------- : 428
CaNAC69 : DLQFPTLRSSSSTSSISIHAKATTS--TTTSTYITPSQDYTSEIDLWNFARSTSSLLSSSEPICHVSNTSV----------------------------------------------- : 427
CaNAC08 : ----HTLRNKCNTN-NQHGAAPSVE--TSAHKFPFHYLGCHSHHDIMKFSK------------------------------------------------------------------- : 339
CaNAC56 : GGDGMNEWGMLDRLVNEDSTKLGGSRFEDANNHQINQLSLRGEMDIWGYGKQ------------------------------------------------------------------ : 358
CaNAC59 : -------CHDHTSSDMALLLLQSSRNEEENKFSSFFTTSPDCDFGICVFEKYINE--------------------------------------------------------------- : 335
CaNAC63 : VLSSFGTNILLPNSDMALMLMQGSRDEGNK----FLNASSDCDIGICVFEK------------------------------------------------------------------- : 366
CaNAC61 : --ASKETSSYCNVDEQIANLLENDESKKSETGQEYATSTSNSSCQIDMWK-------------------------------------------------------------------- : 337
CaNAC32 : -------ANYFSHQNLPLMIPHSHHE---------LQSSNQANHILGCFPDS------------------------------------------------------------------ : 316
CaNAC35 : -TTTTLGGE-NHNNNNNLG-YGGEVNINANPNGNNRYMGMEHCVDLDNYWPSY----------------------------------------------------------------- : 346
CaNAC64 : LSHSQLGND-QSNNDSNIS-ASSSKVLECMPQHNQ----QEQPFNLDSIW-------------------------------------------------------------------- : 324
CaNAC09 : FFFSTAAQP-YHGGDNILSGWGLPEEQKVVDTGGVVNMGGLGSSELDCMWNY------------------------------------------------------------------ : 386
CaNAC13 : --DQEC--DFDVDISSMIYNNEMFQ-----VNQEYS---IGHLDN-GFLWNF------------------------------------------------------------------ : 347
CaNAC22 : --DFSVGRDFDSDISSVVFGRDILQR--WFGNPELSPASTGPVVT-DSLWNF------------------------------------------------------------------ : 363
CaNAC57 : SKETDLTTDMNAETSSVVSNFDMSRR--AFENNHHRPPPAAVDLD-GTLWNY------------------------------------------------------------------ : 430
CaNAC14 : SQDTCLSNDINTDTSSVVSKQEIGRNTTLYEDFECP-SSIGPLSNFESLWNDY----------------------------------------------------------------- : 365
CaNAC25 : SQDTGLSN----DTSSAVSKQDMGRNRALYEDLEGPSSSVAPLSDLECLW-DY----------------------------------------------------------------- : 388
CaNAC38 : ------GADSNAEFRDMSTSTPFNSRLALDASVGIDSWSQPQLV-------------------------------------------------------------------------- : 343
CaNAC26 : ----------NMDLEENSLEDGVAS-NLDAIRFEVDNNNMVLL--------------------------------------------------------------------------- : 291
CaNAC29 : ----------DMDL------DGVASSNLDGMRFEVDNNSMVLL--------------------------------------------------------------------------- : 279
CaNAC11 : ----------NNNNNNNQSLKGSPSLGEGSSESYLSEVGMPHMWNNY----------------------------------------------------------------------- : 314
CaNAC10 : VENELLMFDN---------WDELGS-----VVKFAVDSPSL----------------------------------------------------------------------------- : 308
CaNAC20 : VGDDALANEN---------WDSLRS-----VVQFTFDSSKVY--DDCKYFN------------------------------------------------------------------- : 298
CaNAC15 : YNNVNVPRVDDQDFNIG---DAWKSNLLWDTSFCPCDVPSTY-YSTTKCYT------------------------------------------------------------------- : 406
CaNAC70 : ERERSIGFSYSMPFNVG---DAWKSSLVWDTSSCTSDEPSG--YSTTKCYT------------------------------------------------------------------- : 407
CaNAC12 : YWET-IGRTIEFPFNLSPNYDALRENFTWDSLPCTSEMSTNYVTSVAKCYT------------------------------------------------------------------- : 390
CaNAC36 : PNPNHLSTLLMQPATLN-SSVLPITPIPTNFSDRLWDWNPIPEDHPNPSFK------------------------------------------------------------------- : 418
CaNAC03 : ---------------------------------------------------------------------------------------------------------------------- :   -
CaNAC53 : ---------------------------------------------------------------------------------------------------------------------- :   -
CaNAC51 : ---------------------------------------------------------------------------------------------------------------------- :   -
CaNAC45 : KIGGSGRSASCLEEVIMGCTSSSSDIKEVLLH-KENTIIYLFLTISCGKRERERERERE GTLNFQYCIVFCVMMNE------------------------------------------ : 457
CaNAC65 : KLGIGGRSTSCLEELIMGCTSTTHHIKDKEESSITNSQEAEWLKYSSYWPDPDNP-DHHG---------------------------------------------------------- : 418
CaNAC18 : SVYAQLGPEHLKKDIEDCQNLVLDPANTAEHDAPPSELRLSQLEFSSQDSFIAWGYSKALK--------------------------------------------------------- : 426
CaNAC37 : NVTSTS---------------VLD---TLELDTPP-DFDLSNLTFCSQDSILDWMDRL------------------------------------------------------------ : 394
CaNAC04 : LVFLLIILIITSQFEWKQQLVADVELNPAVSQKQHHISTAEETVKEKIILVQEKNIRRLNELVRHLQEQLQQCKGSNGTINSTVGPLTERILELERQQILED---------------- : 476
CaNAC23 : ---------------------------------------------------------------------------------------------------------------------- :   -
CaNAC60 : NMNFTSQYQLNQYFQAQQMQDSLSTDMASMPQYQPNQHSQAQTDEEYDDPFELIDFDR------------------------------------------------------------ : 298
```

```
                   *       840         *       860         *       880         *       900         *       920         *       940
CaNAC01  : ---------------------------------------------------------------------------------------------------------------------- :    -
CaNAC49  : ---------------------------------------------------------------------------------------------------------------------- :    -
CaNAC48  : ---------------------------------------------------------------------------------------------------------------------- :    -
CaNAC17  : ---------------------------------------------------------------------------------------------------------------------- :    -
CaNAC34  : ---------------------------------------------------------------------------------------------------------------------- :    -
CaNAC28  : ---------------------------------------------------------------------------------------------------------------------- :    -
CaNAC55  : ---------------------------------------------------------------------------------------------------------------------- :    -
CaNAC54  : ---------------------------------------------------------------------------------------------------------------------- :    -
CaNAC41  : INGYEQPRNYNTVISGENGIVRRARPAQDEQWKT---FSMQGTAQRRLRLKKIEDCSNV---TVKDGSCTQEQHNSKPIIPVKKEAAESRAADESATCT---HASKQQKVSLKSIYNI :  549
CaNAC71  : VDGYEQMRNLDTVVQGDTGIRIRTRQRRNEQPITNPVMPAQGSAPRRILLGGFVTHSLVSEETTKDRSSAPVNHNSETIISREKQTSEKHAAGESATDTNINDVDELEKTSLASDDTR :  542
CaNAC02  : ---------------------------------------------------------------------------------------------------------------------- :    -
CaNAC27  : ---------------------------------------------------------------------------------------------------------------------- :    -
CaNAC05  : ---------------------------------------------------------------------------------------------------------------------- :    -
CaNAC50  : ---------------------------------------------------------------------------------------------------------------------- :    -
CaNAC16  : ---------------------------------------------------------------------------------------------------------------------- :    -
CaNAC52  : ---------------------------------------------------------------------------------------------------------------------- :    -
ANAC029  : ---------------------------------------------------------------------------------------------------------------------- :    -
CaNAC24  : ---------------------------------------------------------------------------------------------------------------------- :    -
CaNAC06  : ---------------------------------------------------------------------------------------------------------------------- :    -
CaNAC67  : ---------------------------------------------------------------------------------------------------------------------- :    -
ANAC019  : ---------------------------------------------------------------------------------------------------------------------- :    -
ANAC055  : ---------------------------------------------------------------------------------------------------------------------- :    -
ANAC072  : ---------------------------------------------------------------------------------------------------------------------- :    -
CaNAC40  : ---------------------------------------------------------------------------------------------------------------------- :    -
ATAF1    : ---------------------------------------------------------------------------------------------------------------------- :    -
OsNAC6   : ---------------------------------------------------------------------------------------------------------------------- :    -
CaNAC43  : ---------------------------------------------------------------------------------------------------------------------- :    -
CaNAC47  : ---------------------------------------------------------------------------------------------------------------------- :    -
ATAF2    : ---------------------------------------------------------------------------------------------------------------------- :    -
SNAC1    : ---------------------------------------------------------------------------------------------------------------------- :    -
CaNAC39  : ---------------------------------------------------------------------------------------------------------------------- :    -
CaNAC46  : ---------------------------------------------------------------------------------------------------------------------- :    -
CaNAC21  : ---------------------------------------------------------------------------------------------------------------------- :    -
CaNAC19  : MCLALCFMWASHSN-------------------------------------------------------------------------------------------------------- :  624
CaNAC44  : ISLAICTMWVNHIIASP----------------------------------------------------------------------------------------------------- :  636
CaNAC66  : ---------------------------------------------------------------------------------------------------------------------- :    -
CaNAC07  : ---------------------------------------------------------------------------------------------------------------------- :    -
CaNAC58  : ---------------------------------------------------------------------------------------------------------------------- :    -
CaNAC62  : ---------------------------------------------------------------------------------------------------------------------- :    -
CaNAC30  : ---------------------------------------------------------------------------------------------------------------------- :    -
CaNAC42  : ---------------------------------------------------------------------------------------------------------------------- :    -
CaNAC31  : SATLEPISDLVCSKAAFVLSHSWVFLTGFSVVVLSLSFKIGSLMYTGK---------------------------------------------------------------------- :  578
CaNAC33  : PTNIAAGNDTATVKRAGRKGVSFLFFPILIALCAFLWVSIGNLRLLGRCISP------------------------------------------------------------------ :  558
CaNAC68  : ---------------------------------------------------------------------------------------------------------------------- :    -
CaNAC69  : ---------------------------------------------------------------------------------------------------------------------- :    -
CaNAC08  : ---------------------------------------------------------------------------------------------------------------------- :    -
CaNAC56  : ---------------------------------------------------------------------------------------------------------------------- :    -
CaNAC59  : ---------------------------------------------------------------------------------------------------------------------- :    -
CaNAC63  : ---------------------------------------------------------------------------------------------------------------------- :    -
CaNAC61  : ---------------------------------------------------------------------------------------------------------------------- :    -
CaNAC32  : ---------------------------------------------------------------------------------------------------------------------- :    -
CaNAC35  : ---------------------------------------------------------------------------------------------------------------------- :    -
CaNAC64  : ---------------------------------------------------------------------------------------------------------------------- :    -
CaNAC09  : ---------------------------------------------------------------------------------------------------------------------- :    -
CaNAC13  : ---------------------------------------------------------------------------------------------------------------------- :    -
CaNAC22  : ---------------------------------------------------------------------------------------------------------------------- :    -
CaNAC57  : ---------------------------------------------------------------------------------------------------------------------- :    -
CaNAC14  : ---------------------------------------------------------------------------------------------------------------------- :    -
CaNAC25  : ---------------------------------------------------------------------------------------------------------------------- :    -
CaNAC38  : ---------------------------------------------------------------------------------------------------------------------- :    -
CaNAC26  : ---------------------------------------------------------------------------------------------------------------------- :    -
CaNAC29  : ---------------------------------------------------------------------------------------------------------------------- :    -
CaNAC11  : ---------------------------------------------------------------------------------------------------------------------- :    -
CaNAC10  : ---------------------------------------------------------------------------------------------------------------------- :    -
CaNAC20  : ---------------------------------------------------------------------------------------------------------------------- :    -
CaNAC15  : ---------------------------------------------------------------------------------------------------------------------- :    -
CaNAC70  : ---------------------------------------------------------------------------------------------------------------------- :    -
CaNAC12  : ---------------------------------------------------------------------------------------------------------------------- :    -
CaNAC36  : ---------------------------------------------------------------------------------------------------------------------- :    -
CaNAC03  : ---------------------------------------------------------------------------------------------------------------------- :    -
CaNAC53  : ---------------------------------------------------------------------------------------------------------------------- :    -
CaNAC51  : ---------------------------------------------------------------------------------------------------------------------- :    -
CaNAC45  : ---------------------------------------------------------------------------------------------------------------------- :    -
CaNAC65  : ---------------------------------------------------------------------------------------------------------------------- :    -
CaNAC18  : ---------------------------------------------------------------------------------------------------------------------- :    -
CaNAC37  : ---------------------------------------------------------------------------------------------------------------------- :    -
CaNAC04  : ---------------------------------------------------------------------------------------------------------------------- :    -
CaNAC23  : ---------------------------------------------------------------------------------------------------------------------- :    -
CaNAC60  : ---------------------------------------------------------------------------------------------------------------------- :    -
```

```
                   *        960         *        980         *       1000         *
CaNAC01 : -------------------------------------------------------------------- :    -
CaNAC49 : -------------------------------------------------------------------- :    -
CaNAC48 : -------------------------------------------------------------------- :    -
CaNAC17 : -------------------------------------------------------------------- :    -
CaNAC34 : -------------------------------------------------------------------- :    -
CaNAC28 : -------------------------------------------------------------------- :    -
CaNAC55 : -------------------------------------------------------------------- :    -
CaNAC54 : -------------------------------------------------------------------- :    -
CaNAC41 : KISRR-VTSAGSILGLRDFLLLAKAPFRSKASPKCTLWSSVFVVSASVLVSLAVFTNIWGYIKI---- :  612
CaNAC71 : KISQQHVTTTESILEWKDSLLGRRVCYSSKISSNRAMWSSVLAVSAIVLVSLVLFVNIWGYLRIETAH :  610
CaNAC02 : -------------------------------------------------------------------- :    -
CaNAC27 : -------------------------------------------------------------------- :    -
CaNAC05 : -------------------------------------------------------------------- :    -
CaNAC50 : -------------------------------------------------------------------- :    -
CaNAC16 : -------------------------------------------------------------------- :    -
CaNAC52 : -------------------------------------------------------------------- :    -
ANAC029 : -------------------------------------------------------------------- :    -
CaNAC24 : -------------------------------------------------------------------- :    -
CaNAC06 : -------------------------------------------------------------------- :    -
CaNAC67 : -------------------------------------------------------------------- :    -
ANAC019 : -------------------------------------------------------------------- :    -
ANAC055 : -------------------------------------------------------------------- :    -
ANAC072 : -------------------------------------------------------------------- :    -
CaNAC40 : -------------------------------------------------------------------- :    -
ATAF1   : -------------------------------------------------------------------- :    -
OsNAC6  : -------------------------------------------------------------------- :    -
CaNAC43 : -------------------------------------------------------------------- :    -
CaNAC47 : -------------------------------------------------------------------- :    -
ATAF2   : -------------------------------------------------------------------- :    -
SNAC1   : -------------------------------------------------------------------- :    -
CaNAC39 : -------------------------------------------------------------------- :    -
CaNAC46 : -------------------------------------------------------------------- :    -
CaNAC21 : -------------------------------------------------------------------- :    -
CaNAC19 : -------------------------------------------------------------------- :    -
CaNAC44 : -------------------------------------------------------------------- :    -
CaNAC66 : -------------------------------------------------------------------- :    -
CaNAC07 : -------------------------------------------------------------------- :    -
CaNAC58 : -------------------------------------------------------------------- :    -
CaNAC62 : -------------------------------------------------------------------- :    -
CaNAC30 : -------------------------------------------------------------------- :    -
CaNAC42 : -------------------------------------------------------------------- :    -
CaNAC31 : -------------------------------------------------------------------- :    -
CaNAC33 : -------------------------------------------------------------------- :    -
CaNAC68 : -------------------------------------------------------------------- :    -
CaNAC69 : -------------------------------------------------------------------- :    -
CaNAC08 : -------------------------------------------------------------------- :    -
CaNAC56 : -------------------------------------------------------------------- :    -
CaNAC59 : -------------------------------------------------------------------- :    -
CaNAC63 : -------------------------------------------------------------------- :    -
CaNAC61 : -------------------------------------------------------------------- :    -
CaNAC32 : -------------------------------------------------------------------- :    -
CaNAC35 : -------------------------------------------------------------------- :    -
CaNAC64 : -------------------------------------------------------------------- :    -
CaNAC09 : -------------------------------------------------------------------- :    -
CaNAC13 : -------------------------------------------------------------------- :    -
CaNAC22 : -------------------------------------------------------------------- :    -
CaNAC57 : -------------------------------------------------------------------- :    -
CaNAC14 : -------------------------------------------------------------------- :    -
CaNAC25 : -------------------------------------------------------------------- :    -
CaNAC38 : -------------------------------------------------------------------- :    -
CaNAC26 : -------------------------------------------------------------------- :    -
CaNAC29 : -------------------------------------------------------------------- :    -
CaNAC11 : -------------------------------------------------------------------- :    -
CaNAC10 : -------------------------------------------------------------------- :    -
CaNAC20 : -------------------------------------------------------------------- :    -
CaNAC15 : -------------------------------------------------------------------- :    -
CaNAC70 : -------------------------------------------------------------------- :    -
CaNAC12 : -------------------------------------------------------------------- :    -
CaNAC36 : -------------------------------------------------------------------- :    -
CaNAC03 : -------------------------------------------------------------------- :    -
CaNAC53 : -------------------------------------------------------------------- :    -
CaNAC51 : -------------------------------------------------------------------- :    -
CaNAC45 : -------------------------------------------------------------------- :    -
CaNAC65 : -------------------------------------------------------------------- :    -
CaNAC18 : -------------------------------------------------------------------- :    -
CaNAC37 : -------------------------------------------------------------------- :    -
CaNAC04 : -------------------------------------------------------------------- :    -
CaNAC23 : -------------------------------------------------------------------- :    -
CaNAC60 : -------------------------------------------------------------------- :    -
```

Figure S1. Multiple alignment of 71 CaNACs of chickpea and well-known stress-responsive NACs from *Arabidopsis* (ATAF1/ANAC002, 019, 029, 055, 072 and ATAF2/081) and rice (SNAC1/ONAC002 and OsNAC6/SNAC2/ONAC048). Conserved NAC domain and subdomains (A-E) are indicated by thick blue line and black thin black lines, respectively, above the sequences. The putative nuclear localization signal (NLS) is shown by a blue double-headed arrow below the sequence. Putative stress-related NAC subgroup is highlighted in red-colored background, and membrane-bound CaNAC members are highlighted in turquoise-colored background.
