# Supplementary material for: Genome-Wide Identification and Expression Analysis of the CaNAC Family Members in Chickpea during Development, Dehydration and ABA Treatments
Source: PLoS One. 2014 Dec 5;9(12):e114107. doi: 10.1371/journal.pone.0114107 (PMC4257607; doi:10.1371/journal.pone.0114107)
Supplement: Figure S3 — Expression of 23 selected CaNAC genes in chickpea roots and leaves under dehydration and ABA treatments. (A) Summary of the results of the expression data. (B) Venn diagram analysis of dehydration- and ABA-responsive CaNAC genes in roots and leaves of chickpea plants. The ABA- and/or dehydration-responsive genes were defined as those whose expression is altered by at least 2-fold (P<0.05) at 2 and/or 5 h of dehydration and/or ABA treatment. (PDF) [file pone.0114107.s003.pdf]

A

#	Gene	Dry		ABA		ABA responsiveness
		Leaves	Roots	Leaves	Roots	
1	*CaNAC02*	Down	Down	Down	Down	Dependent
2	*CaNTL1/CaNAC04*	Down	Down	Down	Down	Dependent
3	*CaNAC05*	Up				Independent
4	*CaNAC06*	Up	Up	Up	Up	Dependent
5	*CaNAC16*	Up	Up			Independent
6	*CaNTL2/CaNAC19*	Up	Up			Independent
7	*CaNAC21*	Up			Up	Dependent
8	*CaNAC24*	Down	Up	Up	Up	Dependent
9	*CaNAC27*	Up	Up			Independent
10	*CaNTL3/CaNAC31*					
11	*CaNTL4/CaNAC33*					
12	*CaNAC39*					
13	*CaNAC40*	Up	Up		Up	Dependent
14	*CaNTL5/CaNAC41*	Up				Independent
15	*CaNAC43*	Up	Up		Up	Dependent
16	*CaNTL6/CaNAC44*		Up			Independent
17	*CaNAC46*	Down	Down	Up		Dependent
18	*CaNAC47*	Up	Up			Independent
19	*CaNAC50*	Up	Up	Up	Up	Dependent
20	*CaNAC52*	Up	Up	Up	Up	Dependent
21	*CaNTL7/CaNAC57*	Up		Up		Dependent
22	*CaNAC67*	Up	Up	Up	Up	Dependent
23	*CaNTL8/CaNAC71*					

B

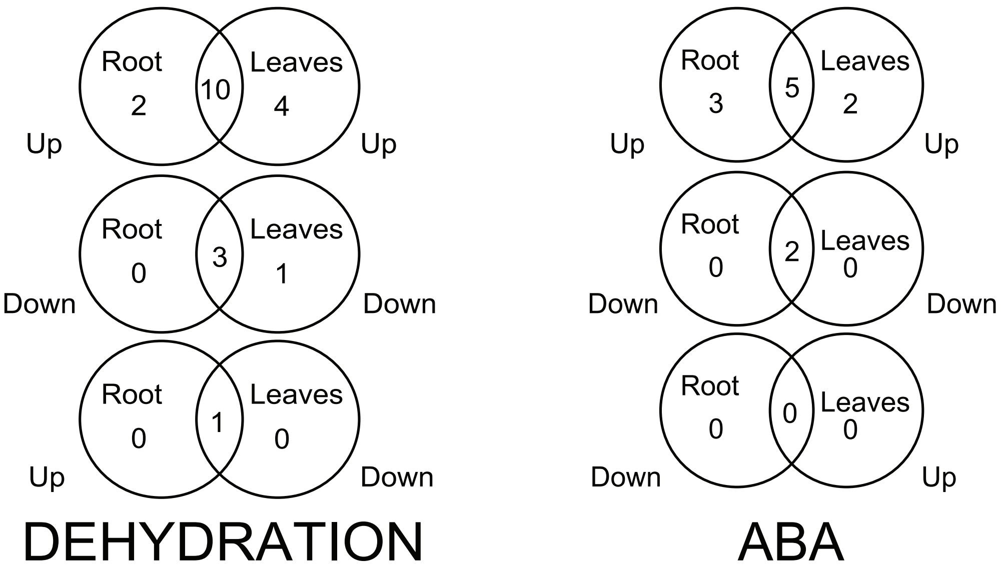

Figure S3. Expression of 23 selected *CaNAC* genes in chickpea roots and leaves under dehydration and ABA treatments. (A) Summary of the results of the expression data. (B) Venn diagram analysis of dehydration- and ABA-responsive CaNAC genes in roots and leaves of chickpea plants. The ABA- and/or dehydration-responsive genes were defined as those whose expression is altered by at least 2-fold ($P < 0.05$) at 2 and/or 5 h of dehydration and/or ABA treatment.
