# Supplementary material for: Genome-Wide Identification and Expression Analysis of the CaNAC Family Members in Chickpea during Development, Dehydration and ABA Treatments
Source: PLoS One. 2014 Dec 5;9(12):e114107. doi: 10.1371/journal.pone.0114107 (PMC4257607; doi:10.1371/journal.pone.0114107)
Supplement: Figure S2 — Phylogenetic relationship of NAC proteins from chickpea (CaNACs) and Arabidopsis (ANACs). The unrooted phylogenetic tree was constructed using the full NAC protein sequences. The membrane-bound CaNAC and ANAC proteins are indicated in blue-colored and green-colored letters, respectively. (PDF) [file pone.0114107.s002.pdf]

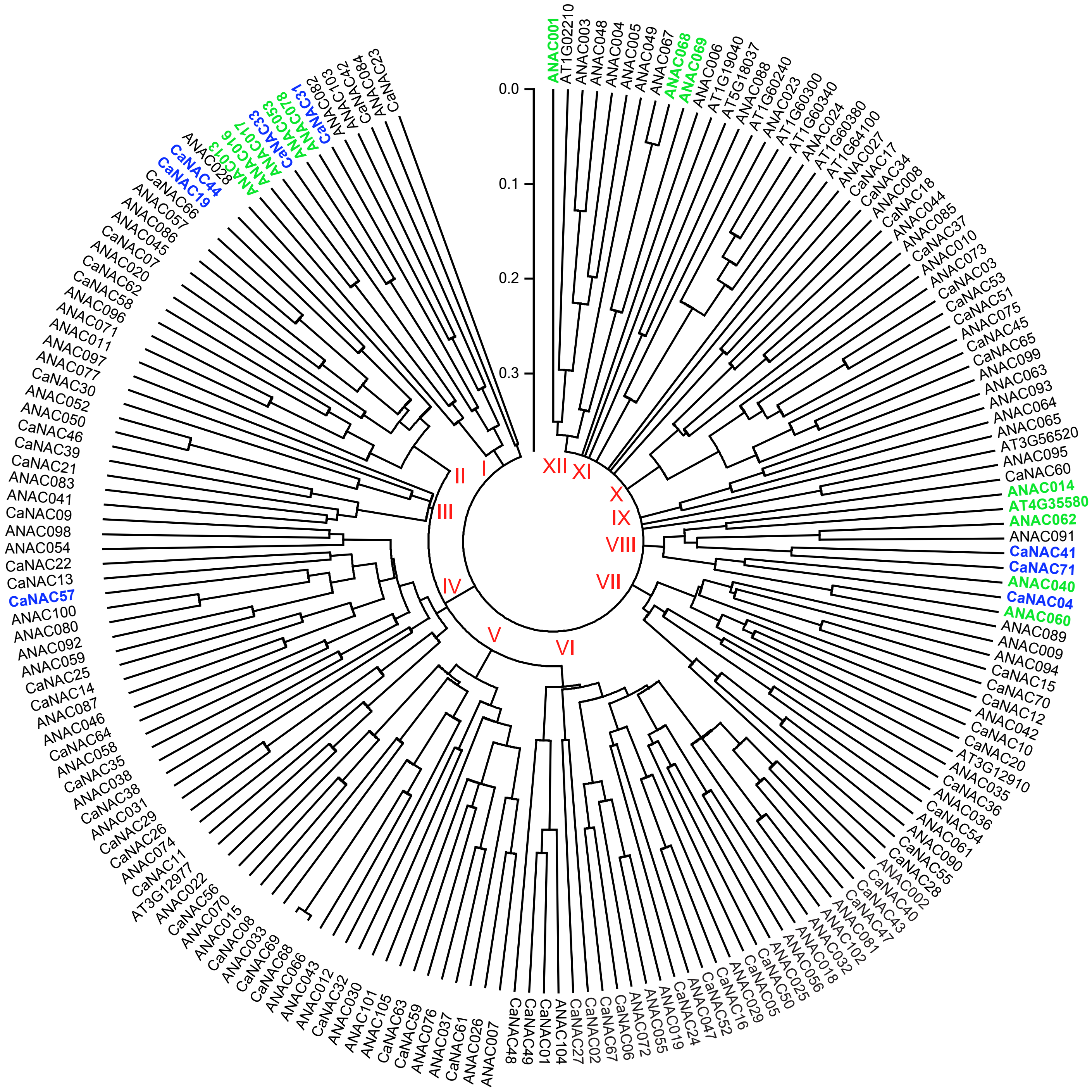

Figure S2. Phylogenetic relationship of NAC proteins from chickpea (CaNACs) and *Arabidopsis* (ANACs). The unrooted phylogenetic tree was constructed using the full NAC protein sequences. The membrane-bound CaNAC and ANAC proteins are indicated in blue-colored and green-colored letters, respectively.
